# Exploratory Analysis of the Innate Immune System in Aortic Stenosis Patients With and Without ASCVD

**DOI:** 10.1016/j.jacbts.2026.101692

**Published:** 2026-10-14

**Authors:** Amber van Broekhoven, Eveline P.M. van Doorn, Wieteke Broeders, Aysun Cetinyurek-Yavuz, Erwin S. Zegers, Mihai G. Netea, Alexander B.A. Vonk, Niels van Royen, Hans W.M. Niessen, Jan H. Cornel, Niels P. Riksen, Saloua El Messaoudi

**Affiliations:** a Department of Cardiology, Radboud University Medical Center, Nijmegen, The Netherlands; b Department of Internal Medicine, Radboud University Medical Center, Nijmegen, The Netherlands; c Department of IQ Health, Radboud University Medical Center, Nijmegen, The Netherlands; d Acibadem Heart Center, Acibadem International Medical Center, Rotterdam, The Netherlands; e Department of Immunology and Metabolism, Life and Medical Sciences Center, University of Bonn, Bonn, Germany; f Amsterdam Cardiovascular Sciences, Amsterdam, The Netherlands; g Department of Cardiac Surgery, Amsterdam University Medical Center, Amsterdam, The Netherlands; h Department of Pathology, Amsterdam University Medical Center, Amsterdam, The Netherlands; i Dutch Network for Cardiovascular Research (WCN), Utrecht, The Netherlands; j Department of Cardiology, Northwest Clinics, Alkmaar, The Netherlands

**Keywords:** aortic stenosis, atherosclerotic cardiovascular disease, innate immune system

## Abstract

Calcific aortic valve disease (CAVD) leads to aortic stenosis (AS) through inflammation and fibro-calcific remodeling. Although CAVD and atherosclerosis share features, not all patients with AS develop atherosclerotic cardiovascular disease (ASCVD), suggesting mechanistic heterogeneity. This case-control study evaluated inflammatory profiles and valvular remodeling in 128 patients with AS (59.4% with ASCVD) and 105 controls. Immune cell composition and monocyte markers were analyzed by flow cytometry, whereas cytokine production was assessed after peripheral blood mononuclear cell (PBMC) stimulation. Both AS groups had higher interleukin (IL)-6, leukocyte, monocyte, and neutrophil counts than controls, with lower monocyte human leukocyte antigen-DR isotype and higher C-C chemokine receptor (CCR)2 expression. Cytokine responses were also elevated, particularly in AS patients with ASCVD. They also showed higher IL-6 concentrations, higher proportions of classical monocytes, and lower intermediate and non-classical monocytes compared with patients without ASCVD. Overall, AS was associated with a pro-inflammatory monocyte phenotype and function, most prominently in patients with ASCVD. ClinicalTrials.gov ID: NCT04717219.

## Highlights

• CAVD and atherosclerosis share many similarities, but heterogeneity exists, as many patients with AS lack ASCVD.
• Systemic inflammation and monocyte activation are consistently present in patients with AS regardless of ASCVD status.
• Future studies should evaluate immunomodulatory therapies across the heterogeneous patient population with AS.

Calcific aortic valve disease (CAVD) is the most common valvular disease in older adults in Western countries. It is characterized by chronic inflammation and progressive fibrocalcific remodeling of the aortic valve, ultimately resulting in valve narrowing and impaired blood flow, known as aortic stenosis (AS).^1^^,^^2^ Without treatment, severe symptomatic AS carries a 2-year mortality rate of 50%.^3^^,^^4^ As the global population ages, AS is expected to place an increasing burden on health care systems.^5^ Currently, no effective pharmacologic treatment exists to slow down or stop the progression of the disease, highlighting a critical unmet need in this growing patient population.

Epidemiological and histopathologic data indicate that CAVD is an active, multifactorial disease sharing key features with atherosclerosis,^6^, ^7^, ^8^ the underlying cause of atherosclerotic cardiovascular disease (ASCVD). Both conditions have overlapping risk factors such as hypertension, hyperlipidemia, and smoking.^9^, ^10^, ^11^ Coronary artery disease (CAD), one of the manifestations of ASCVD, is common in AS, with a reported prevalence ranging from 25%-50%, which increases with age.^12^, ^13^, ^14^, ^15^, ^16^

Although CAD is common in patients with severe AS, a considerable proportion of patients undergoing surgical aortic valve replacement (SAVR) or transcatheter aortic valve replacement show no evidence of concomitant CAD.^17^, ^18^, ^19^ Over 40% of patients with significant aortic valve calcification on computed tomography have minimal coronary artery calcification, and up to 14% have none.^20^ Despite overlapping risk factors and pathophysiology with atherosclerosis, statins fail to improve AS outcomes, suggesting distinct underlying mechanisms.^21^ Together, these findings point to overlap, but also differences between AS and ASCVD pathophysiology.

A previous study suggests distinct AS phenotypes: An atherosclerotic type characterized by inflammation, calcium, and ceroid depositions, and a degenerative type characterized by fibrosis and inflammation without calcification.^22^ Recent evidence highlighted innate immune activation as a contributor to the pathophysiology of AS. We previously reported an altered circulating monocyte phenotype in patients with AS, characterized by higher expression of the C-C chemokine receptor (CCR)2, a higher production of pro-inflammatory cytokines after *ex vivo* peripheral blood mononuclear cell (PBMC) stimulation, and a higher circulating interleukin (IL)-6 concentration.^23^ However, it remains unknown whether and to what extent these immune alterations differ between patients with AS with and without ASCVD.

Therefore, this study aimed to explore whether circulating inflammatory and immune parameters, as well as valve histology, differ between patients with AS with and without concomitant ASCVD.

## Methods

### Data availability

The major resource table is outlined in Supplemental Table 1.

### Study population

This study includes 2 cohorts of patients with tricuspid AS; one cohort has extensive peripheral immunophenotyping data, whereas the other cohort includes valve histological data. Patients were recruited at 3 centers in the Netherlands: Radboud University Medical Center (Radboudumc) in Nijmegen, Canisius Wilhelmina Hospital in Nijmegen, and Amsterdam University Medical Center (Amsterdam UMC) in Amsterdam. ASCVD was defined as a history of atherosclerotic cardiovascular events and/or coronary stenosis ≥30% as determined by coronary angiography.

#### Immunophenotyping cohort

The immunophenotyping cohort included 128 patients with AS and 105 healthy controls, recruited at Radboudumc and the Canisius Wilhelmina Hospital, and has been described before.^23^ In contrast to the previous study, which included only participants with complete immunophenotyping data, the present analysis uses a larger cohort of additional patients and healthy controls. All participants provided written informed consent. The Medical Ethics Committee Region Arnhem-Nijmegen approved the study protocol (NL72973.091.20), and all study procedures and experiments were conducted in line with the Declaration of Helsinki of 1964 and all following amendments. The study was registered at ClinicalTrials.gov ID NCT04717219.

Adult patients with known tricuspid AS or patients who newly presented to the department of cardiothoracic surgery or cardiology with tricuspid AS, defined by transthoracic echocardiography following the 2017 European Society of Cardiology and the European Association for Cardio-Thoracic Surgery Guidelines for the Management of Valvular Heart Disease,^24^ were screened for eligibility. In parallel, healthy controls were recruited via local advertisements and visited the hospital for further screening and a transthoracic echocardiogram to rule out AS.

Exclusion criteria consisted of the use of anti-inflammatory drugs, active auto-inflammatory disease, recent systemic inflammation or vaccination (<1 month before inclusion), recent acute ischemic cardiac event (<3 months before inclusion), history of endocarditis, history of chest radiation therapy, history of bone marrow transplantation, and active malignancy (except for local squamous cell skin carcinoma or local basal cell carcinoma). After obtaining informed consent, venous blood was drawn for deep immunophenotyping and routine laboratory parameters.

#### Valve histology cohort

The valve histology cohort comprised 29 patients with a tricuspid AS who underwent elective SAVR at Radboudumc, with valves collected prospectively. These patients were also included in the immunophenotyping cohort. Seventeen additional excised aortic valves were obtained from the Amsterdam UMC biobank, where they had been stored following routine pathologic examination post-SAVR. Clinical data were extracted from the electronic health records. The collection, storage, and use of patient material and associated clinical data were conducted in accordance with the guidelines of the Ethics Committee of the Amsterdam UMC and the Medical Ethics Committee Region Arnhem-Nijmegen in the Netherlands. All patients provided informed consent for the storage and use of aortic valve specimens combined with related clinical data.

### Blood sample collection

Blood samples were drawn via venous puncture into ethylenediaminetetraacetic acid (EDTA) vacutainers (Becton Dickinson). The hospital laboratory measured creatinine, urate, total cholesterol, high-density lipoprotein cholesterol (HDL-C), triglycerides, glycated hemoglobin (HbA1c), and glucose using standardized methods. The Friedewald equation was employed to calculate low-density lipoprotein cholesterol (LDL-C). The estimated glomerular filtration rate (eGFR) was calculated using the Chronic Kidney Disease Epidemiology Collaboration (CKD-EPI) equation.

### Whole blood composition and circulating plasma markers

The composition of whole blood was analyzed using the Sysmex XN-450 automated hematology analyzer (Sysmex). EDTA vacutainers were centrifuged at 3,800 revolutions per minute (RPM) for 10 minutes at room temperature. The plasma was then collected and stored at −80 °C for further analysis. IL-6 and C-reactive protein (CRP) concentrations were measured in EDTA plasma using commercially available high-sensitivity (hs) IL-6 and CRP enzyme-linked immunosorbent assay (ELISA) kits (R&D Systems).

### Flow cytometry

Circulating leukocytes, with a specific focus on monocytes, were phenotyped using flow cytometry. Fresh EDTA whole blood was stained with mouse monoclonal antibodies against cluster of differentiation (CD)16, human leukocyte antigen-DR isotype (HLA-DR), CD11c, CD3, CD14, CD56, CD19, CD41, CCR2, CD45, CCR5, and CD11b, after erythrocyte lysis. Detailed methodology is provided in the Supplemental Methods.

### Isolation and stimulation of PBMCs

PBMCs were isolated from EDTA whole blood by density gradient centrifugation using Ficoll-Paque Plus (Cytiva, Life Sciences). The isolated PBMCs were resuspended in Roswell Park Memorial Institute 1,640 medium (RPMI; Life Technologies), supplemented with 50 μg/mL gentamicin (Centrafarm), 1 mmol/L pyruvate (Invitrogen), and 2 mmol/L glutamax (Invitrogen). Cell counts and composition were evaluated using the Sysmex analyzer.

To evaluate innate cytokine production by PBMCs, 5 × 10^5^ PBMCs were stimulated for 24 hours in duplicate in round-bottom 96-well plates, using RPMI (negative control), 10 ng/mL lipopolysaccharide (LPS; Sigma-Aldrich) from *Escherichia coli* (purified as previously described^25^), 10 μg/mL tri-palmitoyl-S-glyceryl-cysteine (Pam3Cys) (EMC Microcollections), 1 × 10^6^/mL heat-killed *Candida albicans* conidia (*C albicans*; UC 820, in-house), and 30 μg/mL polyinosinic:polycytidylic acid (Poly I:C; Invivogen). For IL-1β measurement, cells were additionally stimulated with 300 μg/mL monosodium urate crystals (MSU; Sigma-Aldrich), and with 300 μg/mL MSU combined with 10 ng/mL LPS.

For assessment of adaptive cytokine production, 5 × 10^5^ PBMCs were stimulated for 7 days in duplicate in round-bottom 96-well plates using RPMI (negative control), 1 × 10^6^/mL heat-killed *C albicans* (UC 820, in-house), and 1 × 10^6^/mL heat-killed *Escherichia coli* (*E coli*; ATCC 35218), with 10% human pooled serum.

After incubation at 37 °C with 5% CO_2_ for 24 hours or 7 days, the plates were centrifuged, the supernatants collected, and stored at −80 °C for cytokine analysis. Tumor necrosis factor (TNF)-α, IL-1β, IL-6, IL-1 receptor antagonist (IL-1Ra), and IL-10 were measured in the 24-hour supernatants, whereas IL-17, IL-22, and interferon-γ (IFN-γ) levels were measured in the 7-day supernatants using commercially available DuoSet ELISA kits (R&D Systems). The mean of duplicate measurements was used for further analysis.

### Aortic valve processing

Aortic valves from Radboudumc participants undergoing elective SAVR were collected immediately following surgery. Each valve was sectioned vertically through the center of each leaflet, and the resulting tissue samples were fixed overnight in 4% formalin. Subsequently, samples were decalcified in 10% EDTA and embedded in paraffin to generate formalin-fixed paraffin-embedded (FFPE) blocks, following standard hospital protocol.

At Amsterdam UMC, available FFPE aortic valve specimens from patients with moderate or severe tricuspid AS were obtained from the institutional biobank, where they had been archived following routine pathologic processing.

### Histology and immunohistochemistry

Serial cross-sections (5 μm thick) were prepared from FFPE blocks containing aortic valve specimens. Elastin, fibrosis, and major calcifications were identified using Elastica van Gieson staining, whereas proteoglycans were detected using Alcian Blue staining.

Indirect immunohistochemistry was performed on the DAKO Omnis using monoclonal or polyclonal antibodies against CD45 (DAKO GA751, monoclonal 2B11 + PD7/26, FLEX), CD68 (DAKO GA613, monoclonal PG-M1, FLEX), CD3 (DAKO GA503, polyclonal, FLEX), CD20cy (DAKO GA604, monoclonal L26, FLEX), and myeloperoxidase (MPO) (DAKO GA511, polyclonal, FLEX). Detailed methodology is provided in the Supplemental Methods.

### Morphometric and immunohistochemical analysis

Histological and immunohistochemical preparations were imaged using a 3DHISTECH PANNORAMIC 1000 DX. Digital image analyses were additionally performed for histoanatomical characterization and quantification.

For each participant, multiple aortic valve specimens were analyzed. The content of elastin, fibrosis, major calcification, and proteoglycans was quantified as the area (mm^2^) occupied by each component within the tissue sections, using FIJI software (version 1.53t).^26^ Quantifications were normalized to the total area of the analyzed valve specimen (mm^2^) and expressed as a percentage. For each patient, the mean percentage of each component across all analyzed specimens was calculated.

Immunohistochemical staining for CD3, CD20, CD45, CD68, and MPO was performed to assess cell infiltration in the aortic valve specimens. Quantification of positively stained cells was conducted using QuPath (version 0.4.3).^27^ The number of positive cells was normalized to the area (mm^2^) of the corresponding aortic valve section. For each patient, the mean number of immune cells per unit tissue area (mm^2^) was calculated across all analyzed specimens.

All data were analyzed by a single blinded researcher. A subset of measurements was randomly selected and independently verified by a second blinded researcher.

### Statistical analysis

Immune cell profiles and functional parameters were assessed by comparing patients with AS with and without ASCVD and healthy controls. The percentage of fibrosis, elastin, proteoglycans, and calcification, as well as immune cell densities in aortic valves, were compared between patients with AS with and without ASCVD. Correlations between systemic immune profiles and aortic valve histological features were analyzed for the entire AS cohort and stratified by ASCVD status.

Continuous data are presented as mean and SD when normally distributed or median and 25th to 75th percentiles (Q1-Q3) when non-normally distributed. Normality was determined by assessing descriptive statistics and graphical displays, including histograms and Q-Q plots. Categorical variables are presented as frequencies with percentages. Baseline characteristics were compared using an independent samples *t*-test or Mann-Whitney *U* test for continuous variables and a chi-squared test for categorical variables. For categorical variables with small cell counts, Fisher's exact test or the Fisher-Freeman-Halton test was used as an alternative.

Immunological data were compared using univariable linear regression for continuous variables and binary logistic regression for categorical variables. When normality of residuals was not satisfied, log10 transformation was applied. Outliers were explored using graphical displays. The immunological data consist mostly of continuous variables, except for some of the cytokine production capacity data. If cytokine levels were below the level of detection in ≥30% of participants, cytokine levels were analyzed as a binary variable (reaction/no reaction). To investigate whether the differences in inflammatory parameters were independent of traditional risk factors, we adjusted for age, sex, body mass index (BMI), season (cold and warm) and year of blood collection, active smoking status, LDL-C, statin use, glucose, and systolic blood pressure (SBP). The group mean SBP was used to impute a missing value for one healthy control. In certain cases, logistic regression analysis encountered very large standard errors because of sparsity of the data (with multiple categories and a limited number of observations). To address this, covariates were dropped from the model.

A comparative analysis to detect differences at the valvular level was performed using univariable linear regression models, with adjustment for sex.

To correct for multiple testing, the Benjamini-Hochberg correction was employed with a 10% false discovery rate. For the cytokine production data, we applied a less strict multiple testing correction, adjusting only for the number of stimuli, given the correlations in cytokine responses that cluster by stimulus.^28^ The details of each analysis, including the number of participants and specific adjustment methods, are provided in the table legends.

Correlations were assessed using Spearman's correlation coefficient (r). A 2-sided *P* value of <0.05 was considered significant. Statistical analysis was performed using IBM SPSS Version 29.0 (IBM Corp), and graphs were made using R version 4.4.2 (R Core Team, 2024) and GraphPad Prism version 9.5.1 (GraphPad Software).

## Results

### Participant characteristics

#### Immunophenotyping cohort

The immunophenotyping cohort consisted of 128 patients with AS and 105 healthy controls (Table 1, Supplemental Table 2). Among patients with AS, 80 (62.5%) had severe AS and 48 (37.5%) had mild/moderate AS; concomitant ASCVD was present in 76 (59.4%), whereas 52 (40.6%) patients had no evidence of ASCVD. Among patients with concomitant ASCVD, 56 (73.7%) had a history of cardiovascular events, including myocardial infarction, stroke, coronary artery bypass grafting, percutaneous coronary intervention, or peripheral artery revascularization, whereas 20 (26.3%) had stable ASCVD. The mean age of the overall AS cohort was 73.2 ± 6.2 years. Patients with ASCVD were older than those without (74.3 ± 6.2 vs 71.8 ± 6.0 years; *P* = 0.025). They also had lower total cholesterol, LDL-C, and HDL-C levels, probably because of more frequent use of lipid-lowering therapies. Additionally, patients with AS with ASCVD had higher median (Q1-Q3) HbA1c levels (42 [37-49] vs 38 [35-42] mmol/mol; *P* = 0.008) and a higher prevalence of cardiovascular risk factors, including hypertension (73.7% vs 55.8%; *P* = 0.035) and hyperlipidemia (72.4% vs 44.2%; *P* = 0.001). Among patients with concomitant ASCVD, multiple ASCVD manifestations were commonly present: 30.3% had a prior myocardial infarction, 26.3% had experienced a stroke, 10.5% had peripheral artery disease, and 72.4% had ≥30% coronary stenosis on angiography (Supplemental Table 2).

**Table 1:** Baseline Characteristics of Patients With AS With and Without ASCVD and Healthy Controls

		Patients With AS (N = 128)				
	Healthy Controls (N = 105) Mean ± SD/Median [Q1-Q3]/Number (%)	With ASCVD (N = 76) Mean ± SD/Median [Q1-Q3]/Number (%)	Without ASCVD (N = 52) Mean ± SD/Median [Q1-Q3]/Number (%)	*P* Value (With vs Without ASCVD)	*P* Value (With ASCVD vs Healthy Controls)	*P* Value (Without ASCVD vs Healthy Controls)
Demographics						
Age, y	70.6 ± 5.1	74.3 ± 6.2	71.8 ± 6.0	**0.025**	**<0.001**	0.22
Male	50 (47.6)	52 (68.4)	34 (65.4)	0.72	**0.005**	**0.036**
Clinical parameters						
BMI, kg/m^2^	23.8 ± 2.9	27.7 ± 4.3	27.5 ± 4.9	0.78	**<0.001**	**<0.001**
SBP, mm Hg^a^	137 ± 17	141 ± 19	143 ± 20	0.66	0.11	0.062
DBP, mm Hg^a^	77 ± 9	74 ± 9	77 ± 11	0.15	0.064	0.89
Severe AS		47 (61.8)	33 (63.5)	0.85	-	-
Blood measurements						
TC, mmol/L	5.55 ± 0.95	4.36 ± 1.12	5.01 ± 1.13	**0.002**	**<0.001**	**0.002**
LDL-C, mmol/L	3.35 ± 0.87	2.37 ± 0.89	2.93 ± 0.97	**<0.001**	**<0.001**	**0.008**
HDL-C, mmol/L	1.60 [1.33-1.89]	1.19 [1.03-1.52]	1.45 ± 0.36	**0.014**	**<0.001**	**0.007**
TG, mmol/L	1.06 [0.84-1.54]	1.33 [0.97-1.79]	1.38 [0.94-1.90]	0.91	**0.005**	**0.014**
Creatinine, μmol/L	75 ± 15	90 ± 28	84 ± 19	0.15	**<0.001**	**0.002**
eGFR, mL/min/1.73 m^2^	83 [72-88]	71 [57-87]	77 [64-86]	0.23	**0.001**	**0.041**
Uric acid, mmol/L	0.29 ± 0.07	0.36 ± 0.08	0.34 ± 0.08	0.13	**<0.001**	**<0.001**
Glucose, mmol/L	5.28 ± 0.71	6.20 [5.40-7.83]	5.70 [5.20-6.95]	0.12	**<0.001**	**<0.001**
HbA1c, mmol/mol	37 ± 4	42 [37-49]	38 [35-42]	**0.008**	**<0.001**	0.051
Cardiovascular risk factors						
Diabetes mellitus type 2	1 (1.0)	24 (31.6)	9 (17.3)	0.070	**<0.001**	**<0.001**
Hypertension	24 (22.9)	56 (73.7)	29 (55.8)	**0.035**	**<0.001**	**<0.001**
Hyperlipidemia	14 (13.3)	55 (72.4)	23 (44.2)	**0.001**	**<0.001**	**<0.001**
Actively smoking	0 (0.0)	6 (7.9)	6 (11.5)	0.55	**0.005**	**0.001**

Patients with AS with and without ASCVD were compared, and both groups were also compared with healthy controls. *P*-values that were significant are presented in bold.

AS = aortic stenosis; ASCVD = atherosclerotic cardiovascular disease; BMI = body mass index; CKD-EPI = Chronic Kidney Disease Epidemiology Collaboration; DBP = diastolic blood pressure; eGFR = estimated glomerular filtration rate; HbA1c = glycated hemoglobin; HDL-C = high-density lipoprotein cholesterol; LDL-C = low-density lipoprotein cholesterol; Q1-Q3 = first to third quartiles; SBP = systolic blood pressure; TC = total cholesterol; TG = triglycerides.

a AS with ASCVD N = 76, AS without ASCVD N = 52, healthy controls N = 104.

Compared with healthy controls, patients with AS, regardless of ASCVD status, were more often male, had a higher BMI, and lower cholesterol levels, consistent with a higher use of lipid-lowering medications. Patients with AS with ASCVD were older than controls (74.3 ± 6.2 vs 70.6 ± 5.1 years; *P* < 0.001), whereas patients with AS without ASCVD did not differ in age. Furthermore, patients with AS, regardless of ASCVD status, had higher creatinine, uric acid, and glucose levels compared with healthy controls.

#### Valve histology cohort

The valve histology cohort included 46 patients with AS, of whom 30 (65.2%) had concomitant ASCVD, and 16 (34.8%) did not (Table 2). Of the 46 valve specimens, 17 (37.0%) were obtained from the Amsterdam UMC biobank, and 29 (63.0%) were collected postoperatively from patients undergoing SAVR at Radboudumc.

**Table 2:** Baseline Characteristics of Patients With AS With and Without ASCVD in the Valve Histology Cohort

	Patients With AS (N = 46)		
	With ASCVD (N = 30)	Without ASCVD (N = 16)	*P* Value
	Mean ± SD/Median [Q1-Q3]/Number (%)	Mean ± SD/Median [Q1-Q3]/Number (%)	
Demographics			
Age, y	72.6 ± 6.1	72.0 ± 4.8	0.76
Male	19 (63.3)	7 (43.8)	0.20
Clinical parameters			
BMI, kg/m^2^	27.3 ± 3.9	27.2 ± 5.9	0.96
Severe AS	18 (60.0)	14 (87.5)	0.092
Cardiovascular risk factors			
Diabetes mellitus	10 (33.3)	1 (6.3)	0.068
Hypertension	22 (73.3)	8 (50.0)	0.11
Hyperlipidemia	18 (60.0)	7 (43.8)	0.29
Actively smoking	4 (13.3)	1 (6.3)	0.29
Medical history			
Angina pectoris	17 (56.7)	2 (12.5)	**0.004**
Peripheral arterial disease	2 (6.7)	0 (0.0)	0.54
Heart failure			0.71
NYHA functional class I	3 (10.0)	1 (6.3)	
NYHA functional class II	6 (20.0)	6 (37.5)	
NYHA functional class III	11 (36.7)	4 (25.0)	
NYHA functional class IV	1 (3.3)	1 (6.3)	
Atrial fibrillation	8 (26.7)	2 (12.5)	0.46
Myocardial infarction	9 (30.0)	0 (0.0)	**0.015**
Stroke	1 (3.3)	0 (0.0)	1.00
Coronary artery bypass grafting	2 (6.7)	0 (0.0)	0.54
Percutaneous coronary intervention	7 (23.3)	0 (0.0)	0.078
Peripheral artery revascularization	2 (6.7)	0 (0.0)	0.54
Pacemaker implantation	1 (3.3)	0 (0.0)	1.00
Cardiovascular medication use			
Statin	22 (73.3)	7 (43.8)	**0.048**
High-intensity statin	13 (59.1)	3 (42.8)	0.67
Ezetimibe	1 (3.3)	1 (6.3)	1.00
PCSK9 inhibitor	0 (0.0)	0 (0.0)	N/A
Aspirin	20 (66.7)	3 (18.8)	**0.002**
P2Y12 inhibitor	4 (13.3)	0 (0.0)	0.28
DOAC	7 (23.3)	0 (0.0)	0.078
Vitamin K antagonist	3 (10.0)	2 (12.5)	1.00
Diuretics	10 (33.3)	6 (37.5)	0.78
Beta-blocker	25 (83.3)	5 (31.3)	**<0.001**
Calcium antagonist	12 (40.0)	3 (18.8)	0.14
Renin-angiotensin inhibitor	15 (50.0)	6 (37.5)	0.42

Patients with AS with ASCVD were compared with patients with AS without ASCVD. *P*-values that were significant are presented in bold.

DOAC = direct oral anticoagulant; NYHA = New York Heart Association; PCSK9 = proprotein convertase subtilisin/kexin type 9; other abbreviations as in Table 1.

Among patients with AS, 32 (69.6%) had severe AS and 14 (30.4%) had moderate AS preoperatively. Age, sex, and BMI did not differ between patients with AS with and without ASCVD. As expected, patients with both AS and ASCVD used more medications compared with those without ASCVD.

### Circulating inflammatory plasma markers

Within the AS group, patients with ASCVD had higher plasma IL-6 concentrations than those without (3.58 ± 1.94 vs 2.80 ± 1.83 pg/mL; *P* = 0.004). Compared with healthy controls, IL-6 was higher in patients with AS, regardless of ASCVD status (Table 3). After adjustment for risk covariates, this difference was no longer significant in patients with AS without ASCVD compared with healthy controls.

**Table 3:** Inflammatory Markers and Blood Composition in Patients With AS With and Without ASCVD and Healthy Controls

		Patients With AS (N = 128)				AS With ASCVD vs Healthy Controls		AS Without ASCVD vs Healthy Controls	
	Healthy Controls (N = 105)	With ASCVD (N = 76)	Without ASCVD (N = 52)	Unadjusted *P* Value	Adjusted *P* Value	Unadjusted *P* Value	Adjusted *P* Value	Unadjusted *P* Value	Adjusted *P* Value
	Mean ± SD	Mean ± SD	Mean ± SD						
*CRP, mg/L*^a^	1.61 ± 2.32	1.65 ± 1.67	2.12 ± 2.40	0.15	0.53	0.32	0.53	**0.017**	0.81
*IL-6, pg/mL*^b^	2.02 ± 3.01	3.58 ± 1.94	2.80 ± 1.83	**0.004**	**0.030**	**<0.001**	**<0.001**	**<0.001**	0.13
Leukocytes*,* 10^3^/μL	5.39 ± 1.42	6.57 ± 1.50	6.67 ± 2.00	0.73	0.87	**<0.001**	0.12	**<0.001**	0.062
Hemoglobin*,* mmol/L	8.94 ± 0.70	8.82 ± 0.98	9.07 ± 0.92	0.15	0.59	0.36	0.99	0.31	0.40
Platelets*,* 10^3^/μL	248 ± 64	216 ± 56	230 ± 53	0.13	0.34	**<0.001**	0.46	0.096	0.52
Neutrophils*,* 10^3^/μL	3.32 ± 1.12	3.88 ± 1.26	4.06 ± 1.68	0.48	0.50	**0.002**	0.63	**0.001**	0.73
Lymphocytes*,* 10^3^/μL	1.45 ± 0.46	1.81 ± 0.67	1.76 ± 0.57	0.69	0.14	**<0.001**	**<0.001**	**<0.001**	**<0.001**
Monocytes*,* 10^3^/μL	0.47 ± 0.15	0.64 ± 0.17	0.64 ± 0.22	0.89	0.89	**<0.001**	**0.003**	**<0.001**	**<0.001**
*Eosinophils, 10*^3^*/μL*	0.12 ± 0.08	0.20 ± 0.14	0.19 ± 0.18	1.00	0.69	**<0.001**	**<0.001**	**<0.001**	**0.004**
Basophils*,* 10^3^/μL	0.03 ± 0.02	0.04 ± 0.02	0.04 ± 0.02	0.65	0.16	0.10	**0.001**	0.39	0.31
Neutrophils*,* %	61.0 ± 7.8	58.4 ± 9.8	59.5 ± 9.7	0.51	0.20	0.050	**<0.001**	0.32	**0.004**
Lymphocytes*,* %	27.3 ± 6.9	28.1 ± 9.0	27.2 ± 8.3	0.57	0.21	0.50	**0.012**	0.94	0.059
Monocytes*,* %	8.9 ± 2.0	10.0 ± 2.7	9.9 ± 2.5	0.83	0.64	**0.002**	0.11	**0.010**	**0.011**
*Eosinophils, %*	2.2 ± 1.5	3.0 ± 2.0	2.9 ± 2.7	0.39	0.16	**0.003**	**0.001**	0.12	**0.005**
Basophils*,* %	0.6 ± 0.3	0.6 ± 0.3	0.5 ± 0.3	0.54	0.11	0.091	**0.034**	**0.043**	0.89

Linear regression was used to compare patients with AS with and without ASCVD and healthy controls. Comparisons were adjusted for age, sex, body mass index (BMI), low-density lipoprotein cholesterol (LDL-C), statin use, systolic blood pressure (SBP), glucose, year and season of blood collection, and active smoking status. Variables that are log10-transformed for linear regression are displayed in italics. Both unadjusted and adjusted *P*-values are displayed. *P*-values remaining significant after correction for multiple testing are presented in bold.

CRP = C-reactive protein; IL = interleukin; other abbreviations as in Table 1.

a AS with ASCVD N = 76, AS without ASCVD N = 51, healthy controls N = 105.

b AS with ASCVD N = 75, AS without ASCVD N = 51, healthy controls N = 104.

CRP was higher in patients with AS without ASCVD compared with healthy controls (2.12 ± 2.40 vs 1.61 ± 2.32 mg/L; *P* = 0.017; Table 3); this difference was no longer significant after adjustment for risk covariates.

### Circulating immune cell numbers

No significant differences were observed in counts of circulating leukocyte subsets between patients with AS with and without ASCVD (Table 3).

Compared with healthy controls, patients with AS had significantly higher total leukocyte counts, primarily because of higher absolute numbers of neutrophils, lymphocytes, monocytes, and eosinophils. After adjusting for covariates, the difference in neutrophil counts was no longer significant in patients with AS with and without ASCVD. Platelet counts were specifically lower in patients with AS with ASCVD compared with controls (216 ± 56 vs 248 ± 64 × 10^3^/μL; *P* < 0.001), but this difference lost significance after adjustment for covariates (Table 3).

The percentage of neutrophils was lower in patients with AS, whereas the percentages of monocytes and eosinophils were higher compared with controls (Table 3). The percentage of monocytes was no longer significant after adjustment for covariates in patients with AS with ASCVD compared with healthy controls.

### Activation state of circulating monocytes

To assess the phenotype of circulating monocytes, we measured surface expression of multiple monocyte activation markers with flow cytometry. There were no significant differences in monocyte surface markers, including HLA-DR, CCR2, CCR5, CD11b, CD11c, and CD41 between the 2 patient groups (Supplemental Table 3). However, analysis of monocyte subsets showed a higher percentage of classical monocytes in patients with AS with ASCVD (89 ± 4 vs 87 ± 6; *P* = 0.010), whereas those without ASCVD had higher percentages of intermediate (4 ± 3 vs 3 ± 2; *P* = 0.030) and non-classical monocytes (9 ± 4 vs 7 ± 3; *P* = 0.013) (Figure 1, Supplemental Table 3).

**Figure 1:**
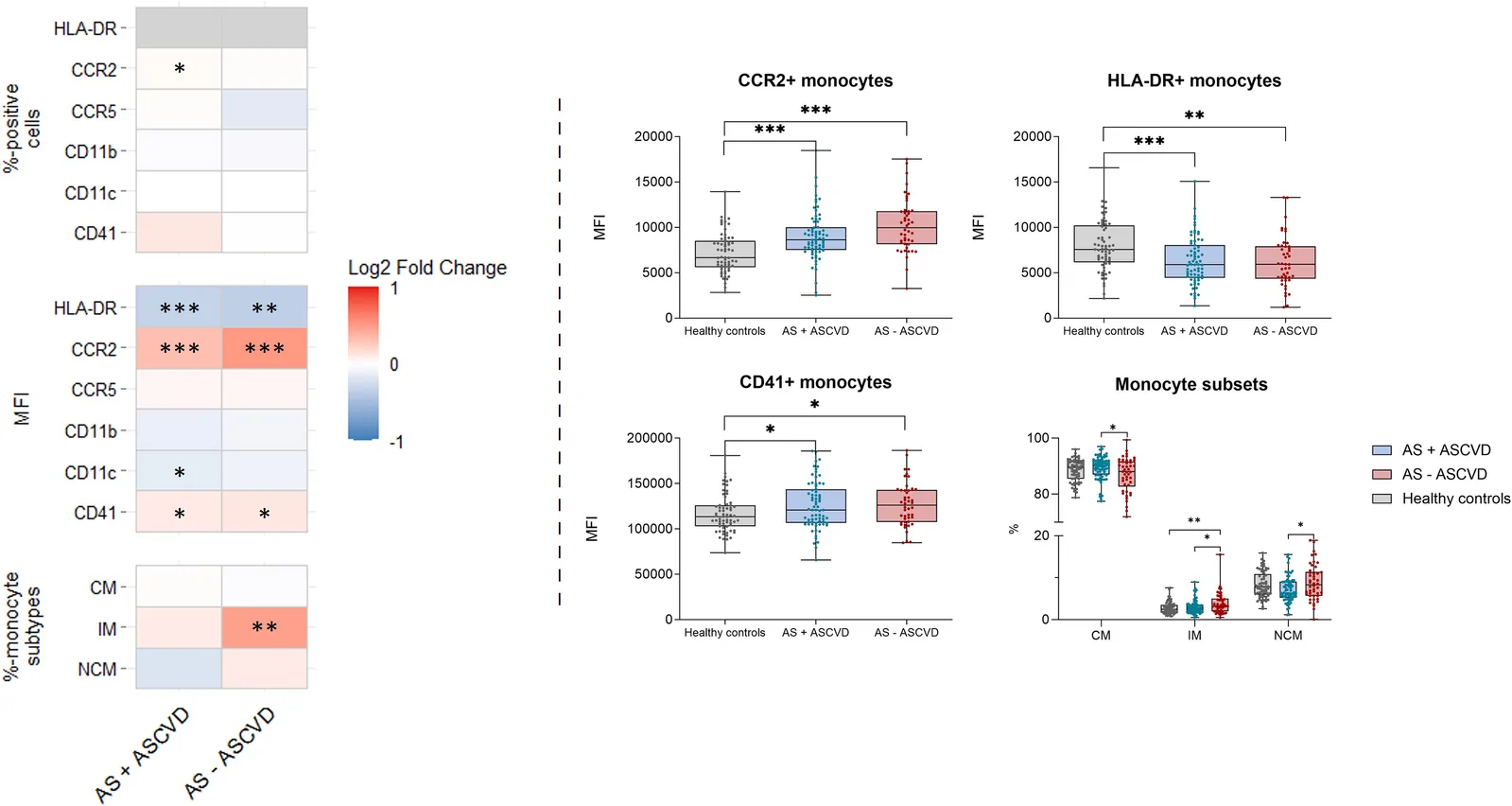
Monocyte Phenotype in Patients With Aortic Stenosis (AS) With and Without Atherosclerotic Cardiovascular Disease (ASCVD) and Healthy Controls Data were analyzed using univariable linear regression and are presented as a heatmap (left panel) and boxplots (right panel). The heatmap illustrates differential expression between patients with AS with and without ASCVD compared with healthy controls. Color intensity reflects the magnitude of difference, represented by base-2 logarithm (log2) fold change, with red indicating higher and blue indicating lower values. Boxplots illustrate differences in monocyte surface marker expression and subsets among both patient groups with AS and healthy controls, presented as median with 25th to 75th percentiles (Q1-Q3). Significant unadjusted *P*-values that remained significant after multiple testing are presented as ∗*P* < 0.05, ∗∗*P* < 0.01, and ∗∗∗*P* < 0.001. CCR2 = C-C motif chemokine receptor 2; CCR5 = C-C motif chemokine receptor 5; CD11b = cluster of differentiation 11b; CD11c = cluster of differentiation 11c; CD41 = cluster of differentiation 41; CM = classical monocytes; HLA-DR = human leukocyte antigen-DR isotype; IM = intermediate monocytes; MFI = median fluorescence intensity; NCM = non-classical monocytes.

Compared with healthy controls, patients with AS, regardless of ASCVD status, exhibited significantly higher CCR2 median fluorescence intensity (MFI) on monocytes (healthy controls: 7,150 ± 2,181 vs AS with ASCVD: 8,985 ± 2,656 and AS without ASCVD: 10,228 ± 2,950; *P* < 0.001) (Figure 1, Supplemental Table 3). HLA-DR MFI was significantly lower compared with controls (healthy controls: 7,923 ± 2,747 vs AS with ASCVD: 6,323 ± 2,602; *P* < 0.001 and AS without ASCVD: 6,219 ± 2,781; *P* = 0.002); this difference lost significance in both patients with AS with and without ASCVD after adjusting for covariates (Figure 1, Supplemental Table 3). Patients with AS with ASCVD showed a higher percentage of CCR2+ monocytes (90 ± 5 vs 89 ± 5; *P* = 0.048) and a lower MFI of CD11c (40,590 ± 8,109 vs 44,877 ± 14,354; *P* = 0.031) compared with controls, and this significance was lost after adjusting for covariates. CD41 MFI was higher in patients with AS regardless of ASCVD status, compared with controls, and this difference became nonsignificant after adjustment (Figure 1, Supplemental Table 3). Regarding monocyte subsets, intermediate monocyte percentages were significantly higher in patients with AS without ASCVD compared with controls (4 ± 3 vs 3 ± 1; *P* = 0.005).

### Ex vivo PBMC cytokine production

No significant differences in PBMC cytokine production were observed between patients with AS with and without ASCVD following *ex vivo* stimulation (Supplemental Table 4).

PBMCs from patients with AS with ASCVD showed higher cytokine production for several cytokines compared with healthy controls following 24-hour stimulation (Figure 2, Supplemental Table 4). Specifically, TNF-α production was higher in response to LPS (427 ± 667 vs 239 ± 323 pg/mL; *P* = 0.019), Pam3Cys (611 ± 742 vs 355 ± 485 pg/mL; *P* = 0.046), *C albicans* (3,657 ± 1,847 vs 2,737 ± 1,980 pg/mL; *P* = 0.006), and Poly I:C (47.9 vs 30.2%; *P* = 0.038). IL-1β production was significantly higher after LPS (3,626 ± 3,314 vs 2,625 ± 2,106 pg/mL; *P* = 0.024), Pam3Cys (4,182 ± 3,000 vs 3,225 ± 2,078 pg/mL; *P* = 0.036), *C albicans* (7,129 ± 2,264 vs 6,071 ± 2,007 pg/mL; *P* = 0.005), and MSU + LPS (4,358 ± 3,789 vs 2,736 ± 2,307 pg/mL; *P* < 0.001) stimulation. Regarding anti-inflammatory cytokines, PBMCs from patients with AS with ASCVD produced significantly higher amounts of IL-1Ra following *C albicans* (21,063 ± 11,270 vs 15,896 ± 6,393 pg/mL; *P* = 0.002) and Poly I:C (12,053 ± 6,290 vs 9,018 ± 5,276 pg/mL; *P* = 0.003) stimulation compared with healthy controls. After adjusting for risk covariates, TNF-α in response to LPS and Poly I:C remained significant.

**Figure 2:**
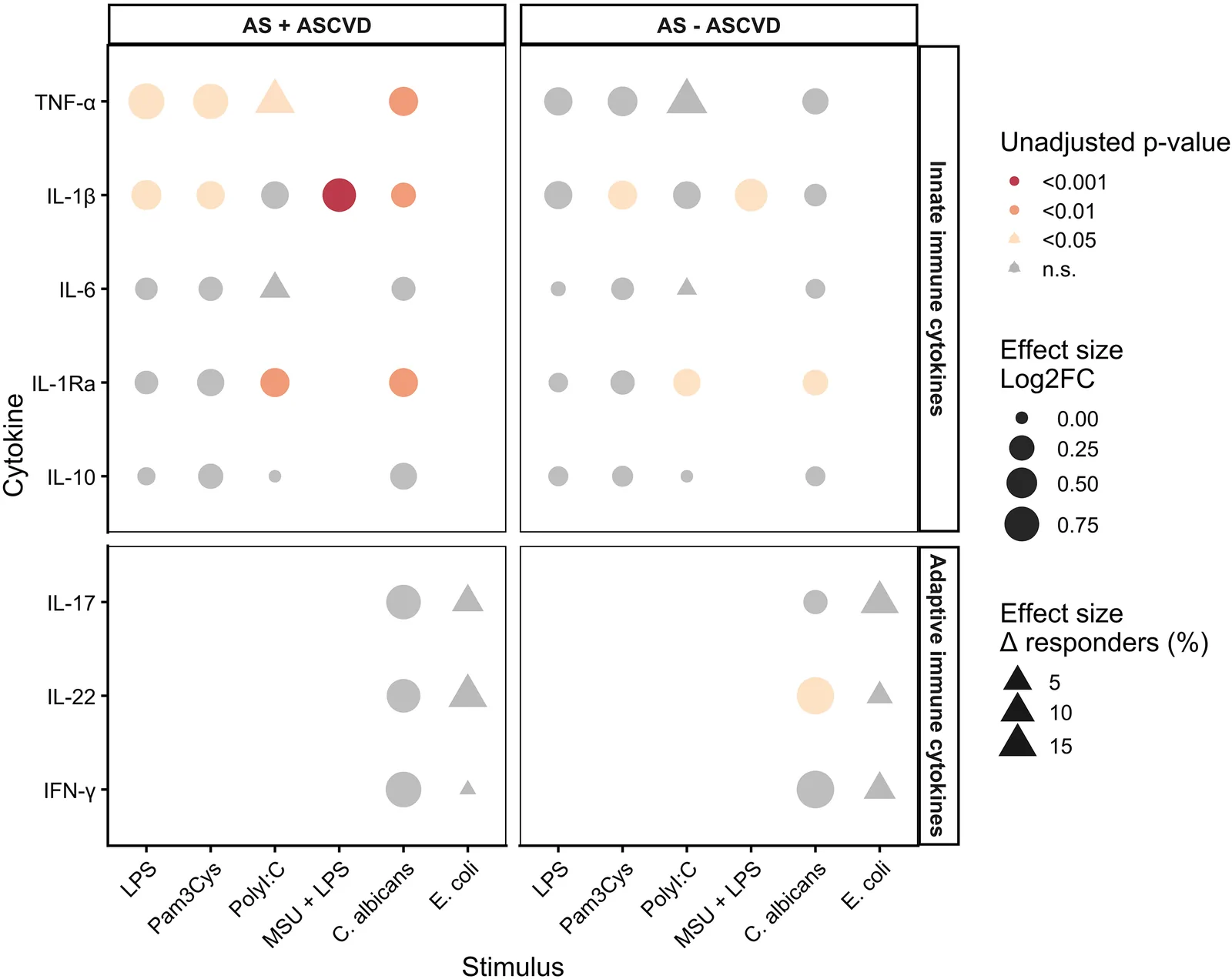
Peripheral Blood Mononuclear Cell (PBMC) Cytokine Production Capacity in Patients With Aortic Stenosis (AS) With and Without Atherosclerotic Cardiovascular Disease (ASCVD) and Healthy Controls Bubble plot showing cytokine production following *ex vivo* PBMC stimulation in patients with AS with and without concomitant ASCVD compared with healthy controls. Cytokine differences were primarily analyzed using linear regression; however, tumor necrosis factor-alpha (TNF-α) and interleukin-6 (IL-6) responses to polyinosinic:polycytidilic acid (Poly I:C), as well as interleukin-17 (IL-17), interleukin-22 (IL-22), and interferon-gamma (IFN-γ) responses to *Escherichia coli*, were assessed using logistic regression. Cytokines are grouped into innate (top panels) and adaptive (bottom panels) immune responses. Circle size represents the magnitude of the base-2 logarithm fold change (log2FC) (effect size), whereas triangle size indicates the percentage difference in responders. Color intensity reflects the statistical significance of unadjusted *P*-values (*P* < 0.001; *P* < 0.01; *P* < 0.05), with grey indicating nonsignificant results. *C albicans = Candida albicans* conidia; IL-1Ra = interleukin-1 receptor antagonist; IL-1β = interleukin-1 beta; IL-10 = interleukin-10; LPS = lipopolysaccharide; MSU = monosodium urate; n.s. = nonsignificant; Pam3Cys = tri-palmitoyl-S-glyceryl-cysteine; other abbreviations as in Figure 1.

In patients with AS without ASCVD compared with healthy controls, a higher IL-1β production was observed in response to Pam3Cys (4,296 ± 2,737 vs 3,225 ± 2,078 pg/mL; *P* = 0.023) and MSU + LPS (4,189 ± 3,365 vs 2,736 ± 2,307 pg/mL; *P* = 0.026). IL-1Ra production in response to *C albicans* (19,054 ± 7,512 vs 15,896 ± 6,393 pg/mL; *P* = 0.021) and Poly I:C (11,386 ± 4,863 vs 9,018 ± 5,276 pg/mL; *P* = 0.019) was also higher than controls (Figure 2, Supplemental Table 4). After adjustment for covariates, only IL-1β in response to Pam3Cys remained significant (*P* = 0.034).

Regarding adaptive immune cytokines, a significantly higher IL-22 in response to *C albicans* (1,507 ± 2,032 vs 791 ± 727 pg/mL; *P* = 0.022) was observed in patients with AS without ASCVD compared with controls, following 7-day stimulation (Figure 2, Supplemental Table 4).

### Valve immunohistology in patients with AS with and without ASCVD

Although overall valvular remodeling phenotype did not differ between patients with AS with and without ASCVD (Figure 3, Supplemental Table 5), relevant numerical differences were evident. Calcification percentages tended to be higher in patients with AS without ASCVD (35.8 ± 12.0 vs 29.4 ± 14.6; *P* = 0.14). In addition, CD68^+^ macrophage content was numerically greater in those with ASCVD (48.9 ± 37.9 vs 29.1 ± 11.7 per mm^2^; *P* = 0.036), but this did not remain significant after multiple testing correction. Consistent with this, other inflammatory cell populations, including CD45^+^, CD20^+^, CD3^+^, and MPO^+^ cells, showed no significant differences between groups (Figure 4, Supplemental Table 5).

**Figure 3:**
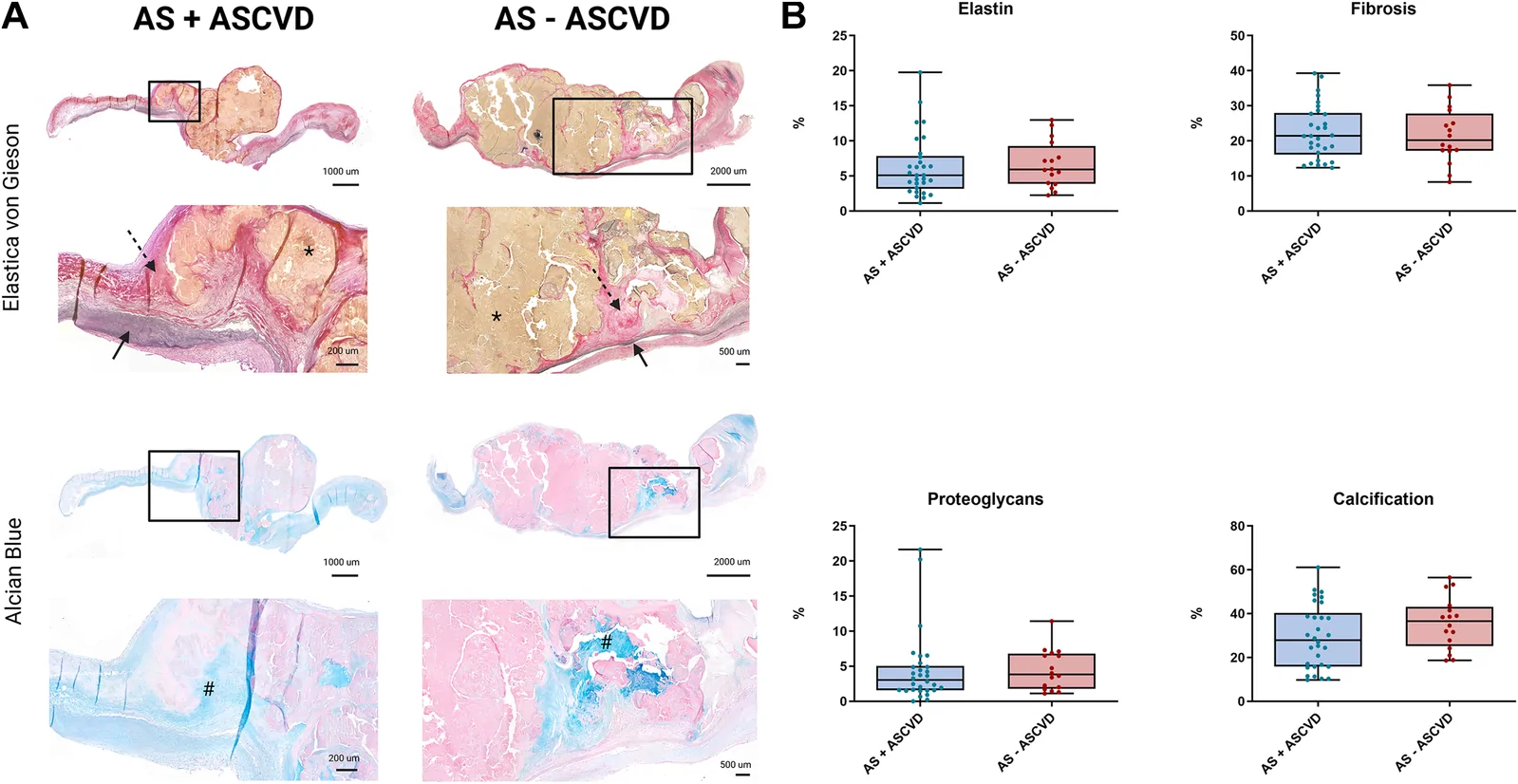
Extracellular Remodeling of Aortic Valve Tissue From Patients With Aortic Stenosis (AS) With and Without Atherosclerotic Cardiovascular Disease (ASCVD) (A) Representative microphotographs of aortic valve sections from patients with AS with and without ASCVD stained with Elastica van Gieson and Alcian Blue. (B) Quantitative analysis of valve remodeling features, presented as boxplots showing the median and 25th to 75th percentiles (Q1-Q3), expressed as the percentage of total aortic valve leaflet tissue on the histological section. Calcification, elastin, and fibrosis were quantified in Elastica van Gieson–stained sections, whereas proteoglycan content was quantified in Alcian Blue–stained sections. For each patient, measurements from all leaflet tissues were averaged. Comparative analyses were performed using univariable linear regression models. Unadjusted *P*-values that remained significant after multiple testing are presented as ∗*P* < 0.05. Arrows indicate elastin; dotted arrows indicate fibrosis; asterisks indicate calcification; hashtags indicate proteoglycans.

**Figure 4:**
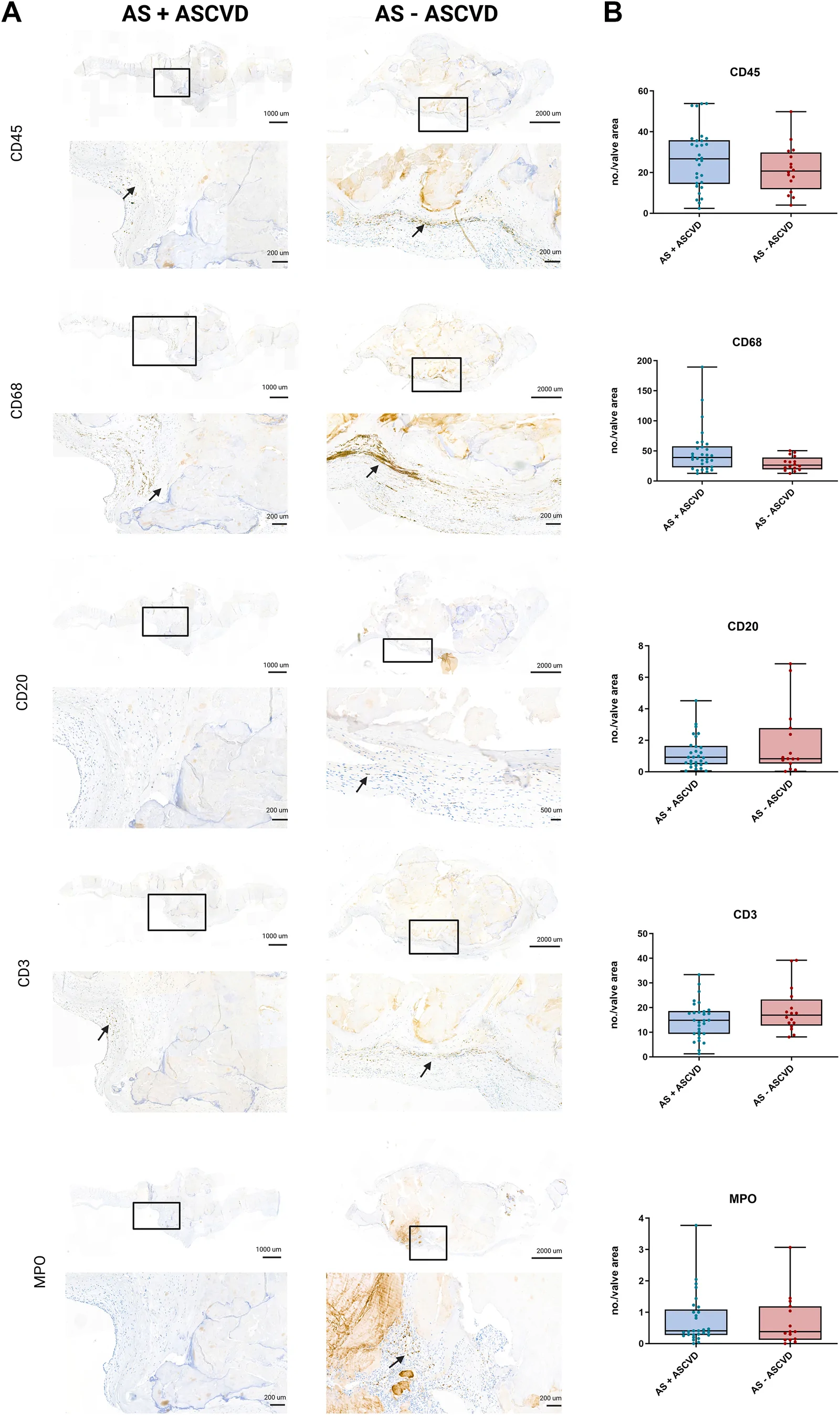
Inflammatory Cell Infiltration in Aortic Valve Tissue From Aortic Stenosis (AS) With and Without Atherosclerotic Cardiovascular Disease (ASCVD) (A) Representative microphotographs of aortic valve sections from patients with AS with and without ASCVD, immunostained for CD45 (pan-leukocyte marker), CD68 (macrophages), CD20 (B-lymphocytes), CD3 (T-lymphocytes), and myeloperoxidase (MPO); neutrophilic granulocytes. (B) The extent of immune cell infiltration is presented as boxplots showing the median and 25th to 75th percentiles (Q1-Q3), quantified as the number of positive cells per mm^2^ of aortic valve leaflet tissue. For each patient, values were averaged across all available valve leaflet tissues. Group comparisons were conducted using linear regression analysis. Significant unadjusted *P*-values that remained significant after multiple testing are presented as ∗*P* < 0.05. Arrows indicate positively stained cells. CD3 = cluster of differentiation 3; CD20 = cluster of differentiation 20; CD45 = cluster of differentiation 45; CD68 = cluster of differentiation 68; other abbreviations as in Figure 1.

### Correlating valve phenotype to circulating inflammatory profiles and function

Finally, we explored the association between circulating inflammatory and immune markers and the tissue composition of the aortic valve. Therefore, we calculated the correlations between histological parameters and circulating immune parameters in all patients from whom these data were available (N = 29; N = 16 with ASCVD; N = 13 without ASCVD). We used unsupervised hierarchical clustering to reveal functional clusters of parameters. This revealed clustering of calcification, CD45, MPO, CD20, and CD3 on the one hand, and of elastin, proteoglycans, fibrosis, and CD68 on the other hand (Figure 5A).

**Figure 5:**
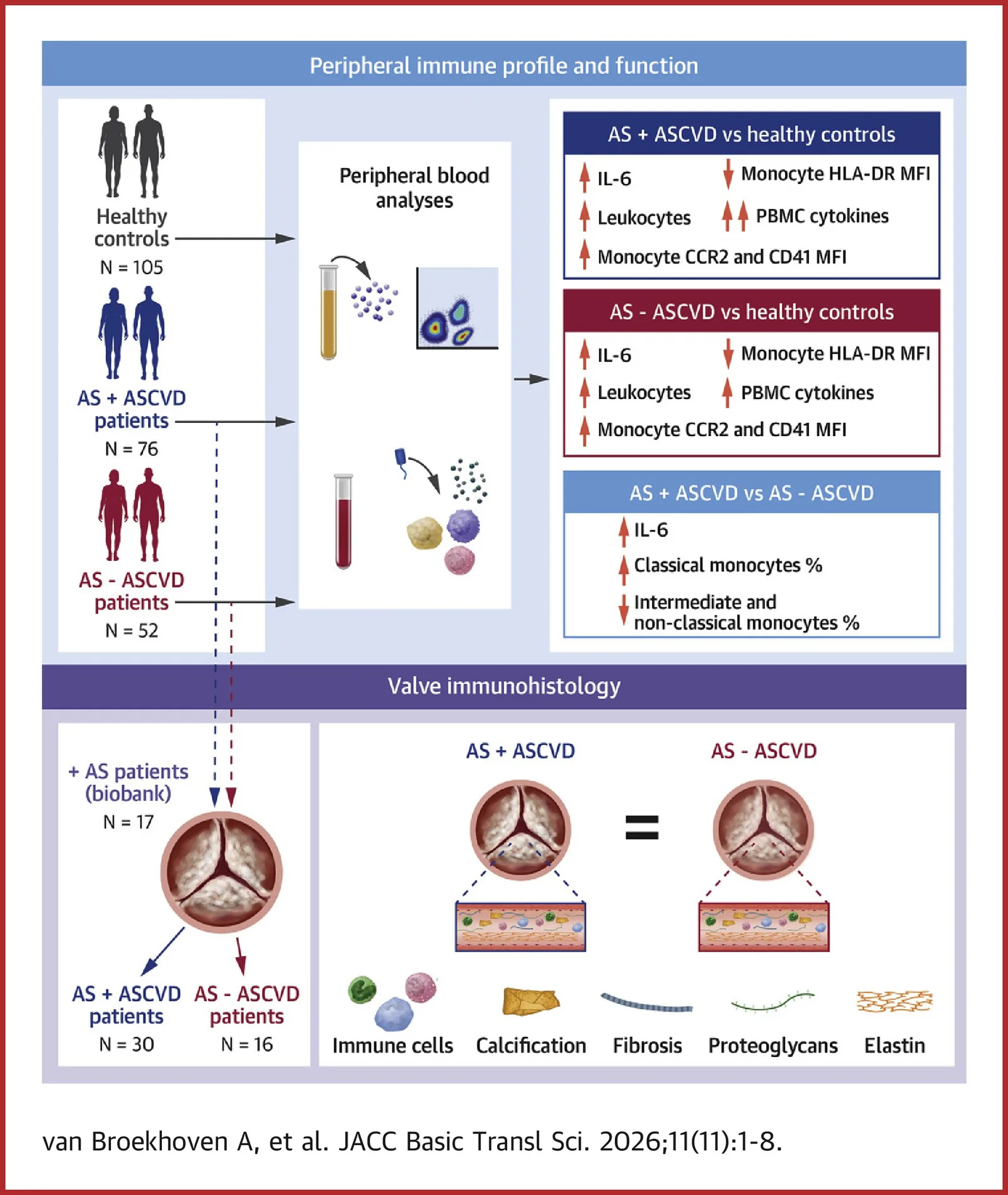
Correlation Patterns of Circulating Inflammatory and Functional Markers and Valve Phenotypical Markers in Patients With Aortic Stenosis (AS) (N = 29) (A) Unsupervised hierarchical clustering heatmap depicts correlations among circulating inflammatory and functional markers and valve phenotypical markers in the total cohort of patients with AS. Clustering was performed using Spearman's correlation coefficients, where red indicates positive correlations and blue indicates negative correlations. Correlations with nominal *P*-values <0.05 are marked with an asterisk (∗). (B) Selected significant correlations are further visualized in accompanying scatterplots. Abbreviations as in Figure 1, Figure 2, Figure 4.

Lymphocyte counts in the blood showed nominally significant positive correlations with CD45^+^ leukocytes (r = 0.445; *P* = 0.015) and CD20^+^ B-lymphocytes (r = 0.461; *P* = 0.012) in the valve (Figure 5B). In contrast, no clear relationship was observed between circulating monocyte numbers or the percentage or MFI of CCR^+^ monocytes and CD68^+^ macrophages in the valve. PBMC IL-6 production upon *ex vivo* LPS stimulation demonstrated a positive correlation with CD68 (r = 0.439; *P* = 0.048).

Another remarkable finding related to PBMC cytokine production capacity is that the lymphocyte-derived cytokines IL-17 and IL-22 mainly show correlations with histological markers. Figure 5B shows scatterplots illustrating IL-22 (*C albicans*) vs elastin and proteoglycans, and IL-17 (*C albicans*) vs fibrosis. In addition, the percentage of proteoglycans was negatively associated with IL-1Ra (LPS) (r = −0.586; *P* = 0.006), IL-1β (Pam3Cys) (r = −0.531; *P* = 0.014), and TNF-α (LPS) (r = −0.521; *P* = 0.017).

We performed a stratified analysis by ASCVD status to explore potential differences in valve remodeling patterns (Supplemental Figure 2). Given the limited sample size in these subgroups, the findings are exploratory and should be interpreted with caution. In patients with AS without ASCVD, we observed similar clustering patterns of histological parameters as the total AS cohort, which were absent in those with ASCVD.

## Discussion

In this study, we set out to test the hypothesis that inflammatory mechanisms underlying AS differ depending on the presence or absence of concomitant ASCVD. Compared with controls, both patients with AS with and without ASCVD showed higher circulating IL-6 concentrations, higher leukocyte, monocyte, eosinophil, and neutrophil counts, and an altered monocyte phenotype (with higher CCR2 and lower HLA-DR expression). The PBMC cytokine production capacity for various cytokines was also higher in patients with AS with and without ASCVD for various stimuli, but this was more pronounced in patients with AS with ASCVD. When comparing patients with AS with and without ASCVD, those with concomitant ASCVD had higher circulating IL-6 concentrations, a higher percentage of classical monocytes, and lower percentages of intermediate and non-classical monocytes. The higher IL-6 and classical monocytes percentage in patients with ASCVD were independent of the difference in classical cardiovascular risk factors. Otherwise, there were no clear differences in leukocyte composition, expression of monocyte activation markers, or PBMC cytokine production capacity between these 2 patient groups.

We recently reported that patients with AS have higher circulating IL-6, higher monocyte counts, and that the monocytes had higher CCR2 and lower HLA-DR expression, as well as higher cytokine production capacity.^23^ The present study builds on these findings by showing that concomitant ASCVD was associated with higher IL-6 concentrations and a predominance of classical monocytes.

Compared with healthy controls, irrespective of the presence of ASCVD, patients with AS exhibited higher plasma IL-6 concentrations. CRP concentrations were higher in patients with AS without ASCVD, whereas there were no differences in CRP concentrations between patients with AS with ASCVD and healthy controls. Within the group of patients with AS, those with ASCVD showed higher plasma IL-6 concentrations than those without ASCVD, whereas no differences in plasma CRP concentrations were observed. These findings might suggest a shift towards IL-6-driven inflammation in AS and especially in the subgroup of patients with AS with ASCVD. This pattern may also be reflected by monocyte HLA-DR MFI^23^: patients with ASCVD, having higher IL-6, tended to exhibit lower HLA-DR MFI, whereas this difference was no longer observed after adjustment for covariates. Previous studies have established that IL-6 plays a central role in the pathogenesis of CAVD by promoting both valvular inflammation and calcification,^29^^,^^30^ with IL-6 gene polymorphisms linked to a higher risk of developing AS.^31^^,^^32^ However, it should be added that several studies failed to detect higher IL-6 concentrations in patients with AS,^32^^,^^33^ which may be attributed to methodological differences, such as the use of regular instead of high-sensitivity assays, such as employed in our study.

Another notable result is that patients with AS with ASCVD have higher classical monocyte percentages compared with patients without ASCVD. Although a previous study has reported an expansion of intermediate monocytes in patients with severe AS compared with healthy controls, classical monocyte proportions often do not differ significantly in AS alone.^33^ In contrast, classical monocytes are well recognized as key contributors to atherosclerotic inflammation, particularly in hypercholesterolemic and inflammatory states, where they show enhanced CCR2-dependent mobilization and preferential recruitment to activated vascular tissue, contributing to early plaque inflammation and disease progression.^34^^,^^35^ Other studies emphasized that ASCVD is characterized by context- and stage-dependent shifts in monocyte subset distributions rather than a uniform monocytic signature, with intermediate and non-classical monocytes often expanded in chronic disease, whereas classical monocytes may predominate in early or highly inflammatory settings.^36^^,^^37^ We propose that the higher classical monocytes in patients with AS with ASCVD could potentially increase valvular inflammation and could contribute to the higher number of valve macrophages in these patients (Figure 4; although this did not reach statistical significance).

Compared with healthy controls, patients with AS with and without ASCVD had significantly higher total leukocyte counts, primarily because of higher absolute numbers of neutrophils, lymphocytes, monocytes, and eosinophils. There were no significant differences in circulating immune cell populations between patients with AS with and without ASCVD. Regarding the activation state of circulating monocytes, both AS subgroups had higher MFI CCR2 expression and lower HLA-DR expression compared with healthy controls. This is in line with our previous study, in which we demonstrated that monocytes from patients with mild/moderate and severe AS exhibit significantly higher CCR2 MFI compared with healthy controls.^23^ CCR2, a chemokine receptor expressed on monocytes, dendritic cells, and valvular interstitial cells, binds ligands such as C-C motif chemokine ligand 2 (CCL2) (monocyte chemoattractant protein-1 [MCP-1]) and CCL7 to mediate monocyte egress from bone marrow and recruitment to inflammatory sites.^38^, ^39^, ^40^, ^41^ Although CCR2-positive monocytes are well recognized in atherogenesis,^42^ data on their role in CAVD remain limited. Notably, calcified aortic valves express higher levels of CCR2 compared with healthy aortic valve tissue.^38^ Moreover, CCL2 is expressed in calcified aortic valves, and this expression is increased by inflammatory stimuli.^43^, ^44^, ^45^ Given its involvement in both atherogenesis and valvular calcification, CCR2 may represent a therapeutic target in patients with AS regardless of the presence of concomitant ASCVD.

Previous studies in ASCVD have shown that monocytes from patients with ASCVD or with risk factors for ASCVD exhibit a pro-inflammatory phenotype, marked by higher PBMC cytokine production.^46^, ^47^, ^48^, ^49^ A recent study from our group reported similar findings in patients with AS compared with healthy controls.^23^ In the current analysis, we specifically demonstrate that patients with AS with concomitant ASCVD exhibit significantly higher cytokine production, primarily of TNF-α and IL-1β, relative to controls, whereas in patients with AS without ASCVD this higher cytokine production capacity is less pronounced. When comparing patients with AS with and without ASCVD, we did not observe differences in PBMC cytokine production.

When studying the valve itself, we observed nonsignificant numerical differences: patients with AS with ASCVD showed higher CD68^+^ macrophage density, whereas those without ASCVD had greater calcification percentages. However, these differences did not reach statistical significance, and overall extracellular remodeling and intra-valvular inflammatory profiles were comparable between groups. The use of broad immunohistochemical markers, combined with the limited sample size, reduces sensitivity. In addition, the surgical valve cohort reflects end-stage disease, whereas the immunophenotyping cohort spans a wider spectrum of disease severity, including mild/moderate and severe AS. Together, these factors may have limited our ability to detect subtle differences and to determine whether a clear distinction exists between degenerative and atherosclerotic valve phenotypes in this cohort.

By analyzing aortic valves from 29 patients from whom we also performed immunophenotyping, we were able to explore potential associations between circulating immune parameters and valve histology. Unsupervised clustering revealed 2 main patterns: calcification clustered with immune cell markers, whereas fibrosis grouped with extracellular matrix components and CD68. This pattern was retained and appeared more coherent in patients with AS without concomitant ASCVD, whereas clustering was less distinct in those with ASCVD. In line with this, in the total AS cohort, specific correlations between circulating immune parameters and valve features were observed, including an association between circulating lymphocytes and valvular CD45^+^ cell infiltration. Moreover, lymphocyte-derived cytokines were linked to extracellular matrix remodeling, with IL-17 correlating with fibrosis and IL-22 with elastin and proteoglycan content. Although these associations were moderate, they support a connection between systemic immune activation and local valve remodeling, which warrants further mechanistic investigation.

In general, our findings support a role for systemic inflammation and innate immune cell activation in the pathophysiology of AS independent of the concomitant presence of ASCVD. For several parameters, such as circulating IL-6, monocyte subsets, and PBMC cytokine production capacity, this is more pronounced in patients with ASCVD. There might be several explanations for the pro-inflammatory profile found in patients with AS. Multiple risk factors for CAVD, such as higher age, hyperlipidemia, hypertension, diabetes mellitus, and obesity, are all associated with higher systemic inflammation.^50^^,^^51^ Our AS population was slightly older, had a higher BMI, had more often hypertension and hyperlipidemia, and were more often smokers compared with healthy controls. However, despite adjustment for these cardiovascular risk factors, several differences compared with healthy controls persisted, indicating that inflammatory markers may play a role independent of these factors.

As expected, patients with AS with ASCVD more often received lipid-lowering therapies, including statins and ezetimibe, and antithrombotic agents, such as acetylsalicylic acid and P2Y12 inhibitors. These drugs are known to exert direct anti-inflammatory or indirect immunomodulatory effects beyond their primary cardiovascular indications, with statins in particular demonstrating well-established direct pleiotropic anti-inflammatory properties.^52^, ^53^, ^54^, ^55^ Antithrombotic agents, including acetylsalicylic acid, may only influence inflammatory pathways indirectly through modulation of platelet activation and platelet-leukocyte interactions.^56^, ^57^, ^58^, ^59^ Taking the influence of statin use into account, the persistence of a pro-inflammatory immune profile in patients with AS with concomitant ASCVD suggests that these pharmacologic interventions only partially mitigate the underlying inflammatory milieu.

Interestingly, whereas our study focused primarily on AS as the index pathology and observed relatively modest immunological differences between patients with AS with and without concomitant ASCVD, a recent study examining immune profiles in patients with CAD, with or without coexisting AS, identified a distinct immune phenotype termed AS–associated CAD.^60^ This AS–associated CAD subgroup exhibited features of immunosenescence and cytotoxicity, contrasting with the heightened immune activation observed in isolated CAD. Although both studies address the interplay between valvular and vascular disease, the differences in immune alterations likely reflect distinct patient populations and perspectives. Our findings suggest a largely shared inflammatory profile in patients with AS regardless of ASCVD status, highlighting systemic inflammation and innate immune activation as contributors to the pathophysiology of AS, although for several parameters this was more pronounced in patients with ASCVD. Conversely, the CAD-focused study suggests a unique immune phenotype in CAD with concomitant AS, possibly driven by the vascular inflammation and immune exhaustion characteristic of advanced CAD pathology. Together, these observations highlight the complexity and heterogeneity of immune remodeling in overlapping cardiovascular diseases. They suggest that the immunopathology may not only depend on the presence of comorbid conditions but also on which cardiovascular disease is primary or predominant in the patient population studied. This emerging paradigm advocates for an immune-based subclassification of cardiovascular diseases, taking into account disease context and comorbidity, which could ultimately enable more tailored immunomodulatory therapeutic strategies beyond traditional anatomical or clinical diagnoses.

Strengths of this study include the amount of clinical data, the inclusion of patients with varying degrees of AS severity, the differentiation between patients with and without known relevant ASCVD, and the in-depth phenotyping of monocytes. In addition, this study was able to correlate circulatory inflammation markers with valve phenotypical parameters.

### Study limitations

This study has several limitations that should be acknowledged. First, the relatively small sample size, particularly after stratification, limited statistical power and may have contributed to the absence of significant differences in valvular histology between groups. In addition, the limited sample size precluded stratified analyses according to severity of AS, which may restrict the generalizability of our findings across the full spectrum of disease severity. Moreover, limited sample sizes constrained meaningful subgroup analyses across ASCVD subcategories, including stratification by obstructive vs nonobstructive CAD, acute ASCVD events vs stable disease, and specific ASCVD manifestations such as stroke or peripheral artery disease. Future studies involving larger, comprehensively phenotyped cohorts, stratified by both ASCVD subtype and AS severity, are warranted to elucidate whether different ASCVD entities differentially influence inflammatory pathways in AS.

Second, the cross-sectional design prevents us from drawing conclusions about causality, and future longitudinal or interventional studies are needed to determine the causality of the observed differences in systemic inflammation and immune phenotype for the development of AS and ASCVD.

Third, data were collected from different cohorts of patients with AS, not all of whom had complete datasets available, which may have impacted the consistency of the findings. In addition, whereas most patients underwent SAVR shortly after blood collection, 3 patients experienced a substantial delay (median 545 days) between sampling and valve collection. This time gap may have introduced biological or clinical changes, potentially confounding correlation analyses. Lastly, the quality of tissue sections also varied because of the presence of calcifications in the aortic valve, potentially influencing the accuracy of histological analyses.

## Conclusions

In conclusion, this study supports a role for systemic inflammation and innate immune cell activation in the pathophysiology of AS, which was more pronounced in patients with AS with concomitant ASCVD. These findings suggest that immune dysregulation contributes to the development of AS, highlighting the potential for targeting immune pathways as a therapeutic strategy.

Perspectives **COMPETENCY IN MEDICAL KNOWLEDGE:** Systemic inflammation and monocyte activation are consistently present in patients with AS regardless of ASCVD status. Although patients with AS with ASCVD had higher IL-6 and distinct monocyte subset distributions, overall circulating immune cell counts and *ex vivo* cytokine responses were similar between groups. Valve histology also revealed no significant differences in inflammatory cell infiltration by ASCVD status. These findings underscore the potential of immune-targeted therapies, irrespective of ASCVD presence.

**TRANSLATIONAL OUTLOOK:** Future studies should focus on validating inflammatory biomarkers and evaluating immune-modulatory treatments for the broader patient population with AS.

### Author contributions

Conceptualization, Prof Dr Riksen, Dr El Messaoudi, Prof Dr van Royen; methodology, Prof Dr Riksen, Dr El Messaoudi, Prof Dr van Royen, Prof Dr Cornel, van Broekhoven, Broeders, Prof Dr Niessen, Prof Dr Netea, Dr Vonk; formal analysis, van Broekhoven, van Doorn, Dr Cetinyurek-Yavuz; investigation, van Broekhoven, van Doorn, Broeders, Zegers; writing, original draft, van Broekhoven, van Doorn; writing, review and editing, all authors; visualization, van Broekhoven, van Doorn; supervision, Prof Dr Riksen, Dr El Messaoudi.

### Data availability

The data supporting this article will be available upon reasonable request to the corresponding author.

## Funding Support and Author Disclosures

This study was conducted with financial support from Radboud University Medical Center. Prof Dr Riksen is further financially supported by a consortium grant from the Dutch Heart Foundation (AtheroNeth; 01-001-2024-0601) and a Vici grant from the Netherlands Organization for Health Research and Development (ZonMw; 09150182410037). All other authors have reported that they have no relationships relevant to the contents of this paper to disclose.
