# Supplementary material for: Exploratory Analysis of the Innate Immune System in Aortic Stenosis Patients With and Without ASCVD
Source: JACC Basic Transl Sci. 2026 Oct 14;11(11):101692. doi: 10.1016/j.jacbts.2026.101692 (PMC13694428; doi:10.1016/j.jacbts.2026.101692)
Supplement: Supplementary file 1 [file mmc1.docx]

**Exploratory analysis of the innate immune system in aortic stenosis patients with and without ASCVD**

Amber van Broekhoven, MD^a*^; Eveline P.M. van Doorn, MSc^a,b*^; Wieteke Broeders, MD^b^; Aysun Cetinyurek-Yavuz, PhD^c^; Erwin S. Zegers, MD^d^; Mihai G. Netea, PhD^b,e^; Alexander B.A. Vonk, MD, PhD^f,g^; Niels van Royen, MD, PhD^a^; Hans W.M. Niessen, MD, PhD^f,h^; Jan H. Cornel, MD, PhD^a,i,j^; Niels P. Riksen, MD, PhD^b^; Saloua El Messaoudi, MD, PhD^a^.

Address for correspondence: Saloua El Messaoudi, Department of Cardiology, Radboud University Medical Center, Geert Grooteplein Zuid 10, 6525 GA Nijmegen, The Netherlands. Email: [Saloua.ElMessaoudi@radboudumc.nl](mailto:Saloua.ElMessaoudi@radboudumc.nl). Tel: +31 24 361 4533

^a^ Department of Cardiology, Radboud University Medical Center, Nijmegen, The Netherlands

^b^ Department of Internal Medicine, Radboud University Medical Center, Nijmegen, The Netherlands

^c^ Department of IQ Health, Radboud University Medical Center, Nijmegen, The Netherlands

^d^ Acibadem Heart Center, Acibadem International Medical Center, Rotterdam, The Netherlands

^e^ Department of Immunology and Metabolism, Life and Medical Sciences Center, University of Bonn, Bonn, Germany

^f^ Amsterdam Cardiovascular Sciences, Amsterdam, The Netherlands

^g^ Department of Cardiac Surgery, Amsterdam University Medical Center, Amsterdam, The Netherlands

^h^ Department of Pathology, Amsterdam University Medical Center, Amsterdam, The Netherlands

^i^ Dutch Network for Cardiovascular Research (WCN), Utrecht, The Netherlands

^j^ Department of Cardiology, Northwest Clinics, Alkmaar, The Netherlands

*Shared first authorship

**Supplementary contents**

[Supplemental Methods	2](#_Toc229934830)

[Flow cytometry	2](#_Toc229934831)

[Histology and immunohistochemistry	2](#_Toc229934832)

[Supplemental Tables	3](#_Toc229934833)

[Supplemental Table 1. Major resource table summarizing the principal reagents, antibodies, and experimental tools used in the study.	3](#_Toc229934834)

[Supplemental Table 2. Extended baseline characteristics of AS patients with and without ASCVD and healthy controls.	6](#_Toc229934835)

[Supplemental Table 3. Monocyte phenotype in AS patients with and without ASCVD and healthy controls.	9](#_Toc229934836)

[Supplemental Table 4. PBMC cytokine production after 24 hours and 7 days of *ex vivo* stimulation in AS patients with and without ASCVD and healthy controls.	10](#_Toc229934837)

[Supplemental Table 5. Histological and immunohistochemical analysis of valvular inflammation and remodeling between AS patients with and without ASCVD.	13](#_Toc229934838)

[Supplemental Figures	14](#_Toc229934839)

[Supplemental Figure 1. Manual gating strategy using flow cytometry.	16](#_Toc229934840)

[Supplemental Figure 2. Correlation patterns of circulating inflammatory and functional profiles and valve phenotypical markers in AS patients with (N=16) and without ASCVD (N=13).	18](#_Toc229934841)

[References	18](#_Toc229934842)

# **Supplemental Methods**

## **Flow cytometry**

Circulating leukocytes, with a particular focus on monocytes, were phenotyped using flow cytometry. Fresh ethylenediaminetetraacetic acid (EDTA) whole blood was mixed with lysis buffer (Becton Dickinson, Franklin Lakes, USA) and incubated at room temperature for 15 minutes in the dark, followed by centrifugation at 1500 revolutions per minute (RPM) to remove erythrocytes. To minimize non-specific binding, the samples were blocked for 10 minutes with Human Trustain FcX blocking agent (Biolegend, San Diego, USA) prior to staining. The cell suspension was stained for 30 minutes at room temperature in the dark with a mix of mouse monoclonal antibodies targeting cluster of differentiation (CD)16, human leukocyte antigen-DR isotype (HLA-DR), CD11c, CD3, CD14, CD56, CD19, CD41, C-C chemokine receptor (CCR)2, CD45, CCR5, and CD11b, as detailed in the Major Resource Table. Distinct leukocyte populations, monocyte subsets, and specific surface proteins were assessed using the Cytoflex cytometer (Beckman Coulter, Brea, USA; RRID: SCR_0127217). Data were processed using FlowJo software (Becton Dickinson, version 10.8.0) employing a manual gating strategy (**Supplemental Figure 1**).

## **Histology and immunohistochemistry**

Cross sections were deparaffinized and rehydrated using Clearify Clearing Agent (DAKO GC810) through incubation for 10-60 seconds at 25°C. Antigen retrieval was performed by EnVision FLEX TRS High pH (DAKO GV800) incubation at 97°C 30 minutes for CD20, CD45, CD68, and myeloperoxidase (MPO) and Envision FLEX TRS Low pH (DAKO GV805) incubation at 97°C 30 minutes for CD3. Primary antibody incubation was then performed for 10 minutes (CD45), 12.30 minutes (CD20), 20 minutes (CD68, MPO), or 30 minutes (CD3). Endogenous enzyme blocking was performed on all tissue sections using EnVision FLEX peroxidase blocking reagent (DAKO GV823) for 3 minutes. For the immunohistochemical staining of CD3, the slides were incubated with EnVision FLEX+ Rabbit Linker (DAKO GV809) for 10 minutes. The endogenous enzyme blocking of CD20, CD45 and CD68 was followed with an incubation of EnVision FLEX+ Mouse Linker (DAKO GV821) for 10 minutes. Subsequently, all sections were visualized by using EnVision FLEX/HRP (DAKO GV825) incubation for 20 minutes and EnVision FLEX Substrate working solution DAB+ Chromogen (DAKO GV825) incubation for 5 minutes. Counterstaining was performed with Hematoxylin (DAKO GC808) incubation for 3 minutes. Between each step the slides were washed using 20x Wash Buffer (DAKO GC807).

# **Supplemental Tables**

## **Supplemental Table 1. Major resource table summarizing the principal reagents, antibodies, and experimental tools used in the study.**

*(Immuno)histochemical staining measurements*

| **Description** | **Vendor or source** | **Catalog#** |
| --- | --- | --- |
| 4% formalin (w/w) | Milestone | In-house |
| 10% EDTA | Milestone | In-house |
| Elastica von Gieson | In-house |  |
| Alcian Blue | In-house |  |
| Polyclonal rabbit anti-human CD3 antibody | DAKO | GA503 |
| Monoclonal mouse anti-human CD20cy antibody | DAKO | GA604 |
| Monoclonal mouse anti-human CD45 antibody | DAKO | GA751 |
| Monoclonal mouse anti-human CD68 antibody | DAKO | GA613 |
| Polyclonal rabbit anti-human MPO antibody | DAKO | GA511 |
| Clearify clearing agent | DAKO | GC810 |
| EnVision FLEX TRS High pH | DAKO | GV800 |
| EnVision FLEX TRS Low pH | DAKO | GV805 |
| EnVision FLEX peroxidase blocking reagent | DAKO | GV823 |
| EnVision FLEX+ Rabbit Linker | DAKO | GV809 |
| EnVision FLEX+ Mouse Linker | DAKO | GV821 |
| EnVision FLEX/HRP | DAKO | GV825 |
| EnVision FLEX Substrate working solution DAB+ chromogen | DAKO | GV825 |
| Hematoxylin | DAKO | GC808 |
| Washing buffer | DAKO | GC807 |

*Flow cytometry measurements*

| **Target antigen** | **Fluorochrome** | **Clone** | **Vendor or Source** | **Catalog#** | **Working concentration** |
| --- | --- | --- | --- | --- | --- |
| Anti-human CD16 monoclonal antibody | FITC | 3G8 | Biolegend | 302006 | 1 : 50 |
| Anti-human HLA-DR monoclonal antibody | PE | Immu-357 | Beckman Coulter | IM1639 | 5 : 50 |
| Anti-human CD11c monoclonal antibody | PEDazzle 594 | Bu15 | Biolegend | 337228 | 1 : 50 |
| Anti-human CD3 monoclonal antibody | PC5.5 | UCHT1 | Biolegend | 300430 | 1 : 50 |
| Anti-human CD14 monoclonal antibody | PC7 | 61D3 | eBioscience | 25-0149-42 | 1 : 50 |
| Anti-human CD56 monoclonal antibody | APC | N901 | Beckman Coulter | IM2474 | 2 : 50 |
| Anti-human CD19 monoclonal antibody | AF700 | HIB19 | Biolegend | 302226 | 1 : 50 |
| Anti-human CD41 monoclonal antibody | APC-Cy7 | HIP8 | Biolegend | 303716 | 1 : 50 |
| Anti-human CD192/CCR2 monoclonal antibody | BV421 | 48607 | Becton Dickinson | 564067 | 2 : 50 |
| Anti-human CD45 monoclonal antibody | BV510 | HI30 | Biolegend | 304036 | 1 : 50 |
| Anti-human CD195/CCR5 monoclonal antibody | BV650 | 3A9 | Becton Dickinson | 564999 | 5 : 50 |
| Anti-human CD11b monoclonal antibody | BV785 | ICRF44 | BioLegend | 301346 | 1 : 50 |
| Brilliant stain buffer |  |  | BD Bioscience | 563794 | 10 : 50 |
| Human TruStain FcX |  |  | Biolegend | 422302 |  |
| BD Pharm Lyse Lysing buffer |  |  | BD Bioscience | 555899 | 6 mL per 600 uL whole blood |
| EDTA |  |  | Life Technologies | 15575020 | 2 mM |

*Peripheral blood mononuclear cell (PBMC) stimulations*

| **Description** | **Vendor or source** | **Sex** | **Catalog#** | **Working concentration** |
| --- | --- | --- | --- | --- |
| PBMCs | Participants study | M, F |  |  |
| Lipopolysaccharide (purified from *Escherichia coli*), 055:B5 | Sigma-Aldrich |  | L2880 | 10 ng/mL |
| Pam3Cys | EMC Microcollections |  | L2000 | 10 µg/mL |
| *Candida albicans* conidia, UC 820 | In-house |  | In-house | 1 x 10^6^/mL |
| Polyinosinic:polycytidylic acid HMW | Bio-connect |  | tlrl-pic-5 | 30 µg/mL |
| Monosodium urate crystals | Sigma-Aldrich |  | In-house | 300 µg/mL |
| *Escherichia coli*, 35218 | ATCC |  | In-house | 1 x 10^6^/mL |

*ELISA antibodies*

| **Target antigen** | **Vendor or Source** | **Catalog#** | **Working concentration** |
| --- | --- | --- | --- |
| High-sensitivity CRP ELISA | R&D Systems | DY1707 | 2 ng/mL |
| High-sensitivity IL-6 ELISA | R&D Systems | HS600C | 20 pg/mL |
| Human IL-1β Duoset ELISA | Bio-Techne (R&D) | DY201 | 2 µg/mL |
| Human TNF-α Duoset ELISA | Bio-Techne (R&D) | DY210 | 2 µg/mL |
| Human IL-6 Duoset ELISA | Bio-Techne (R&D) | DY206 | 1 µg/mL |
| Human IL-1Ra Duoset ELISA | Bio-Techne (R&D) | DY280 | 5 µg/mL |
| Human IL-10 Duoset ELISA | Bio-Techne (R&D) | DY217B | 2 µg/mL |
| Human IL-17 Duoset ELISA | Bio-Techne (R&D) | DY317 | 4 µg/mL |
| Human IL-22 Duoset ELISA | Bio-Techne (R&D) | DY782 | 2 µg/mL |
| Human IFN-γ Duoset ELISA | Bio-Techne (R&D) | DY285B | 2 µg/mL |

*Other*

| **Description** | **Vendor or source** | **Catalog#** |
| --- | --- | --- |
| EDTA K2 vacutainers | Becton Dickinson | 367525 |
| Roswell Park Memorial Institute 1640 Dutch-modified culture medium | Life Technologies | 22409031 |
| Ficoll-paque^TM^ Plus | VWR | 17-1440-03P |
| Pyruvate | Invitrogen | 11360088 |
| Gentamycin | Centrafarm | In-house |
| Glutamax | Invitrogen | 35050087 |
| Round bottom 96-wells plate | Corning | 3359 |
| Phosphate-buffered saline (PBS) | Westburg | LO BE17-516F |
| BSA | Sigma-Aldrich | A9576-50mL |
| VersaComp antibody capture bead kit | Beckman Coulter | B22804 |

## **Supplemental Table 2. Extended baseline characteristics of AS patients with and without ASCVD and healthy controls.**

|  | **Healthy controls  (N=105)** | | **Patients with AS (N=128)** | | | **P-value  (with ASCVD versus healthy controls)** | **P-value  (without ASCVD versus healthy controls)** |
| --- | --- | --- | --- | --- | --- | --- | --- |
|  |  |  | **with ASCVD  (N= 76)** | **without ASCVD (N=52)** | **P-value  (with versus without ASCVD)** |  |  |
|  | **Mean ± SD / Median [Q1-Q3]/ number (%)** | | **Mean ± SD/ Median [Q1-Q3]/ number (%)** | **Mean ± SD/ Median [Q1-Q3]/ number (%)** |  |  |  |
| **Echocardiography** | | | | | | | |
| Aortic valve area (cm^2^)^a^ |  | | 1.00 [0.77-1.20] | 0.86 [0.70-1.21] | 0.92 | - | - |
| MPG (mmHg)^b^ |  | | 32 [20-41] | 39 [24-53] | **0.015** | - | - |
| Vmax (m/s)^c^ |  | | 3.66 ± 0.79 | 4.00 ± 0.97 | **0.043** | - | - |
| Dimensionless index^d^ |  | | 0.28 ± 0.09 | 0.28 ± 0.10 | 0.85 | - | - |
| LVEF (%)^e^ |  | | 53 ± 9 | 54 ± 7 | 0.40 | - | - |
| LVH^f^ |  | | 34 (44.7) | 28 (53.8) | 0.77 | - | - |
| **Coronary angiography** | | | | | | | |
| CAG stenosis^g^ | 1 (1.0) | | 58 (76.3) | 30 (57.7) | **<0.001** | **<0.001** | **<0.001** |
| No / wall irregularities | 1 (1.0) | | 3 (3.9) | 30 (57.7) |  |  |  |
| 30-49% stenosis | 0 (0.0) | | 8 (10.5) | 0 (0.0) |  |  |  |
| 50-69% stenosis | 0 (0.0) | | 5 (6.6) | 0 (0.0) |  |  |  |
| >70% stenosis | 0 (0.0) | | 42 (55.3) | 0 (0.0) |  |  |  |
| **History of cardiovascular events** | | | | | | | |
| Myocardial infarction | 0 (0.0) | | 23 (30.3) | 0 (0.0) | **<0.001** | **<0.001** | N/A |
| Stroke | 0 (0.0) | | 20 (26.3) | 0 (0.0) | **<0.001** | **<0.001** | N/A |
| Coronary artery bypass grafting | 0 (0.0) | | 13 (17.1) | 0 (0.0) | **0.002** | **<0.001** | N/A |
| Percutaneous coronary intervention | 0 (0.0) | | 33 (43.4) | 0 (0.0) | **<0.001** | **<0.001** | N/A |
| Peripheral artery revascularization | 0 (0.0) | | 5 (6.6) | 0 (0.0) | 0.080 | **0.012** | N/A |
| **Medical history** | | | | | | | |
| Angina pectoris | | 0 (0.0) | 19 (25.0) | 6 (11.5) | 0.059 | **<0.001** | **0.001** |
| Peripheral artery disease | | 0 (0.0) | 8 (10.5) | 0 (0.0) | **0.021** | **<0.001** | N/A |
| Heart failure | | 0 (0.0) | 47 (61.8) | 28 (53.8) | 0.27 | **<0.001** | **<0.001** |
| NYHA II | | 0 (0.0) | 20 (26.3) | 18 (34.6) |  |  |  |
| NYHA III | | 0 (0.0) | 21 (27.6) | 8 (15.4) |  |  |  |
| Atrial fibrillation | | 0 (0.0) | 22 (28.9) | 4 (7.7) | **0.003** | **<0.001** | **0.011** |
| Pacemaker implantation | | 0 (0.0) | 9 (11.8) | 2 (3.8) | 0.20 | **<0.001** | 0.11 |
| Rheumatic fever | | 0 (0.0) | 0 (0.0) | 0 (0.0) | N/A | N/A | N/A |
| **Medication use** | | | | | | | |
| Lipid lowering drugs | | 6 (5.7) | 61 (80.3) | 18 (34.6) | **<0.001** | **<0.001** | **<0.001** |
| Statin | | 6 (5.7) | 56 (73.7) | 17 (32.7) | **<0.001** | **<0.001** | **<0.001** |
| Ezetimibe | | 0 (0.0) | 12 (15.8) | 2 (3.8) | **0.033** | **<0.001** | 0.11 |
| PCSK9 inhibitor | | 0 (0.0) | 2 (2.6) | 0 (0.0) | 0.51 | 0.18 | N/A |
| Fibrate | | 0 (0.0) | 2 (2.6) | 0 (0.0) | 0.51 | 0.18 | N/A |
| APT/anticoagulants | | 2 (1.9) | 71 (93.4) | 14 (26.9) | **<0.001** | **<0.001** | **<0.001** |
| Acetylsalicylic acid | | 2 (1.9) | 31 (40.8) | 9 (17.3) | **0.005** | **<0.001** | **<0.001** |
| P2Y12 inhibitor | | 0 (0.0) | 24 (31.6) | 1 (1.9) | **<0.001** | **<0.001** | 0.33 |
| DOAC | | 0 (0.0) | 22 (28.9) | 2 (3.8) | **<0.001** | **<0.001** | 0.11 |
| Vitamin K antagonist | | 0 (0.0) | 7 (9.2) | 2 (3.8) | 0.31 | **0.002** | 0.11 |
| Diuretic | | 6 (5.7) | 29 (38.2) | 19 (36.5) | 0.85 | **<0.001** | **<0.001** |
| Loop diuretic | | 0 (0.0) | 15 (19.7) | 6 (11.5) | 0.22 | **<0.001** | **0.001** |
| Thiazide | | 6 (5.7) | 10 (13.2) | 13 (25.0) | 0.087 | 0.082 | **<0.001** |
| Potassium-sparing | | 0 (0.0) | 9 (11.8) | 2 (3.8) | 0.20 | **<0.001** | 0.11 |
| Betablocker | | 5 (4.8) | 48 (63.2) | 14 (26.9) | **<0.001** | **<0.001** | **<0.001** |
| Calcium channel blocker | | 5 (4.8) | 27 (35.5) | 7 (13.5) | **0.006** | **<0.001** | 0.063 |
| RAAS inhibitor | | 13 (12.4) | 42 (55.3) | 21 (40.4) | 0.098 | **<0.001** | **<0.001** |
| Antidiabetic drugs | | 1 (1.0) | 22 (28.9) | 7 (13.5) | **0.040** | **<0.001** | **0.002** |
| Metformin | | 1 (1.0) | 17 (22.4) | 7 (13.5) | 0.21 | **<0.001** | **0.002** |
| SU-derivate | | 0 (0.0) | 11 (14.5) | 2 (3.8) | 0.051 | **<0.001** | 0.11 |
| GLP-1 agonist | | 0 (0.0) | 0 (0.0) | 1 (1.9) | 0.41 | N/A | 0.33 |
| DPP4-inhibitor | | 0 (0.0) | 1 (1.3) | 0 (0.0) | 1.00 | 0.42 | N/A |
| SGLT2-inhibitor | | 0 (0.0) | 1 (1.3) | 0 (0.0) | 1.00 | 0.42 | N/A |
| Insulin | | 0 (0.0) | 9 (11.8) | 1 (1.9) | **0.048** | **<0.001** | 0.33 |

AS patients with and without ASCVD were compared, and both groups were also compared with healthy controls. P-values that were significant are presented in bold. (APT) antiplatelet therapy, (AS) aortic stenosis, (ASCVD) atherosclerotic cardiovascular disease, (CABG) coronary artery bypass grafting, (CAG) coronary angiography, (DOAC) direct oral anticoagulant, (DPP-4) dipeptidyl peptidase-4, (GLP-1) glucagon-like peptide-1, (LVEF) left ventricular ejection fraction, (LVH) left ventricular hypertrophy, (MPG) mean pressure gradient, (NYHA) New York Heart Association, (PAD) peripheral artery disease, (PCI) percutaneous coronary intervention, (PCSK9) proprotein convertase subtilisin/kexin type 9, (RAAS) renin-angiotensin-aldosterone system, (SGLT2) sodium-glucose cotransporter 2, (SU) sulfonylurea, (Vmax) maximum velocity. ^a^ AS with ASCVD N=68, AS without ASCVD N=49. ^b^ AS with ASCVD N=69, AS without ASCVD N=52. ^c^ AS with ASCVD N=72, AS without ASCVD N=50. ^d^ AS with ASCVD N=64, AS without ASCVD N=44. ^e^ AS with ASCVD N=75, AS without ASCVD N=51. ^f^ AS with ASCVD N=72, AS without ASCVD N=51. ^g^ AS with ASCVD N=58, AS without ASCVD N=30, Healthy controls N=1.

## **Supplemental Table 3. Monocyte phenotype in AS patients with and without ASCVD and healthy controls.**

|  | **Healthy controls (N=65)** | **Patients with AS (N=118)** | | | | **AS with ASCVD  versus Healthy controls** | | **AS without ASCVD versus  Healthy controls** | |
| --- | --- | --- | --- | --- | --- | --- | --- | --- | --- |
|  |  | **with ASCVD (N=72)** | **without ASCVD (N=46)** | **Unadjusted P-value** | **Adjusted P-value** | **Unadjusted P-value** | **Adjusted P-value** | **Unadjusted P-value** | **Adjusted P-value** |
|  | **Mean ± SD** | **Mean ± SD** | **Mean ± SD** |  |  |  |  |  |  |
| **All monocytes** | | | | | | | | | |
| HLA-DR+ Mo (MFI)^a^ | 7923 ± 2747 | 6323 ± 2602 | 6219 ± 2781 | 0.84 | 0.79 | **<0.001** | 0.42 | **0.002** | 0.12 |
| CCR2+ Mo (% gated) | 88.7 ± 5.0 | 90.4 ± 5.2 | 89.7 ± 4.4 | 0.45 | 0.22 | **0.048** | 0.50 | 0.26 | 0.43 |
| CCR2+ Mo (MFI) | 7150 ± 2181 | 8985 ± 2656 | 10228 ± 2950 | 0.019 | 0.22 | **<0.001** | **<0.001** | **<0.001** | **<0.001** |
| CCR5+ Mo (% gated)^b^ | 24.2 ± 17.3 | 24.4 ± 15.2 | 21.7 ± 13.2 | 0.33 | 0.22 | 0.93 | 0.10 | 0.43 | 0.44 |
| CCR5+ Mo (MFI)^b^ | 1473 ± 278 | 1519 ± 173 | 1523 ± 179 | 0.90 | 0.66 | 0.25 | 0.032 | 0.29 | 0.068 |
| CD11b+ Mo (% gated) | 83.1 ± 13.1 | 81.3 ± 12.6 | 80.8 ± 13.7 | 0.83 | 0.86 | 0.42 | 0.76 | 0.37 | 0.28 |
| CD11b+ Mo (MFI) | 16927 ± 5912 | 15543 ± 4263 | 16039 ± 4410 | 0.54 | 0.39 | 0.12 | 0.90 | 0.39 | 0.81 |
| CD11c+ Mo (% gated) | 100 ± 0 | 100 ± 0 | 100 ± 0 | N/A | N/A | N/A | N/A | N/A | N/A |
| CD11c+ Mo (MFI) | 44877 ± 14354 | 40590 ± 8109 | 42167 ± 10804 | 0.37 | 0.58 | **0.031** | 0.77 | 0.28 | 0.38 |
| CD41+ Mo (% gated) | 3.9 ± 1.3 | 4.3 ± 1.8 | 3.9 ± 1.3 | 0.22 | 0.23 | 0.20 | 0.47 | 0.92 | 0.97 |
| CD41+ Mo (MFI) | 116984 ± 20762 | 125781 ± 26128 | 127313 ± 24108 | 0.75 | 0.28 | **0.032** | 0.26 | **0.017** | 0.42 |
| **Monocyte subsets** | | | | | | | | | |
| Classical monocytes (%) | 88.6 ± 3.9 | 89.4 ± 4.1 | 87.0 ± 6.1 | **0.010** | **0.009** | 0.22 | 0.77 | 0.091 | 0.15 |
| Intermediate monocytes (%) | 2.8 ± 1.4 | 3.0 ± 1.6 | 3.9 ± 2.6 | **0.030** | **0.006** | 0.35 | 0.30 | **0.005** | **0.005** |
| Non-classical monocytes (%) | 8.3 ± 3.1 | 7.2 ± 3.0 | 8.9 ± 4.3 | **0.013** | **0.024** | 0.041 | 0.87 | 0.38 | 0.56 |

Linear regression was used to compare AS patients with and without ASCVD and each of the groups to healthy controls, adjusting for age, sex, body mass index (BMI), year and season of blood collection, active smoking status, low-density lipoprotein-cholesterol (LDL-C), statin use, systolic blood pressure (SBP), and glucose. Both unadjusted and adjusted p-values are displayed. P-values remaining significant after multiple testing correction are shown in bold. (AS) aortic stenosis, (ASCVD) atherosclerotic cardiovascular disease, (CCR) C-C chemokine receptor, (CD) cluster of differentiation, (HLA-DR) human leukocyte antigen-DR isotype, (MFI) median fluorescence intensity, (Mo) monocyte. ^a^ AS with ASCVD N=71, AS without ASCVD N=46, Healthy controls N=65. ^b^ AS with ASCVD N=70, AS without ASCVD N=45, Healthy controls N=61.

## **Supplemental Table 4. PBMC cytokine production after 24 hours and 7 days of *ex vivo* stimulation in AS patients with and without ASCVD and healthy controls.**

|  | | **Healthy controls (N=63)** | **Patients with AS**  **(N=116)** | | | | **AS with ASCVD versus  Healthy controls** | | **AS without ASCVD versus Healthy controls** | |
| --- | --- | --- | --- | --- | --- | --- | --- | --- | --- | --- |
|  |  |  | **With ASCVD**  **(N=71)** | **Without ASCVD (N=45)** | **Unadjusted P-value** | **Adjusted P-value** | **Unadjusted P-value** | **Adjusted P-value** | **Unadjusted P-value** | **Adjusted P-value** |
| **Cytokine** | **Stimulus** | **Mean ± SD/ number (%)** | **Mean ± SD/**  **number (%)** | **Mean ± SD/**  **number (%)** |  |  |  |  |  |  |
| **Innate immune system** | | | | | | | | | | |
| TNF-α  (pg/ml) | RPMI | 39 ± 0 | 39 ± 1 | 39 ± 0 | N/A | N/A | N/A | N/A | N/A | N/A |
|  | *LPS* | 239 ± 323 | 427 ± 667 | 311 ± 320 | 0.69 | 0.78 | **0.019** | **0.026** | 0.070 | 0.39 |
|  | *Pam3Cys* | 355 ± 485 | 611 ± 742 | 485 ± 476 | 0.96 | 0.78 | **0.046** | 0.25 | 0.058 | 0.087 |
|  | C. albicans | 2737 ± 1980 | 3657 ± 1847 | 3335 ± 1920 | 0.37 | 0.76 | **0.006** | 0.12 | 0.12 | 0.51 |
|  | Poly I:C | 19 (30.2) | 34 (47.9) | 22 (48.9) | 0.92 | 0.44 | **0.038** | **<0.001** | 0.050 | 0.030 |
| IL-1β  (pg/ml) | RPMI | 20 ± 2 | 21 ± 9 | 25 ± 15 | N/A | N/A | N/A | N/A | N/A | N/A |
|  | *LPS* | 2625 ± 2106 | 3626 ± 3314 | 3393 ± 2456 | 0.67 | 0.69 | **0.024** | 0.29 | 0.13 | 0.78 |
|  | Pam3Cys | 3225 ± 2078 | 4182 ± 3000 | 4296 ± 2737 | 0.84 | 0.91 | **0.036** | 0.25 | **0.023** | **0.034** |
|  | C. albicans | 6071 ± 2007 | 7129 ± 2264 | 6762 ± 2223 | 0.39 | 0.71 | **0.005** | 0.81 | 0.094 | 0.84 |
|  | MSU^a^ | 20 ± 2 | 21 ± 7 | 21 ± 5 | N/A | N/A | N/A | N/A | N/A | N/A |
|  | *MSU + LPS*^a^ | 2736 ± 2307 | 4358 ± 3789 | 4189 ± 3365 | 0.49 | 0.54 | **<0.001** | 0.11 | **0.026** | 0.42 |
|  | *Poly I:C^b^* | 62 ± 70 | 79 ± 92 | 79 ± 77 | 0.98 | 0.94 | 0.13 | 0.034 | 0.18 | **0.003** |
| IL-6  (pg/ml) | RPMI | 47 ± 1 | 50 ± 19 | 52 ± 22 | N/A | N/A | N/A | N/A | N/A | N/A |
|  | *LPS* | 3804 ± 2431 | 4241 ± 2766 | 3779 ± 2298 | 0.25 | 0.48 | 0.23 | 0.71 | 0.95 | 0.030 |
|  | Pam3Cys | 8701 ± 4874 | 10077 ± 7141 | 9729 ± 5326 | 0.78 | 0.85 | 0.20 | 0.96 | 0.30 | 0.33 |
|  | *C. albicans* | 2514 ± 1211 | 2889 ± 1600 | 2656 ± 1671 | 0.21 | 0.51 | 0.15 | 0.46 | 0.96 | 0.48 |
|  | Poly I:C | 43 (68.3) | 53 (74.6) | 31 (68.9) | 0.50 | 0.14 | 0.41 | 0.044 | 0.94 | 0.50 |
| IL-1Ra  (pg/ml) | RPMI | 5379 ± 5147 | 6834 ± 5553 | 6989 ± 6094 | N/A | N/A | N/A | N/A | N/A | N/A |
|  | LPS | 14442 ± 7271 | 16493 ± 7597 | 15221 ± 8367 | 0.40 | 0.86 | 0.11 | 0.82 | 0.61 | 0.98 |
|  | *Pam3Cys* | 11655 ± 6293 | 14654 ± 9533 | 13483 ± 9136 | 0.41 | 0.96 | 0.058 | 0.55 | 0.41 | 0.93 |
|  | C. albicans | 15896 ± 6393 | 21063 ± 11270 | 19054 ± 7512 | 0.29 | 0.89 | **0.002** | 0.51 | **0.021** | 0.19 |
|  | Poly I:C | 9018 ± 5276 | 12053 ± 6290 | 11386 ± 4863 | 0.55 | 0.86 | **0.003** | 0.74 | **0.019** | 0.55 |
| IL-10  (pg/ml) | RPMI | 35 ± 0 | 35 ± 0 | 36 ± 4 | N/A | N/A | N/A | N/A | N/A | N/A |
|  | *LPS* | 207 ± 166 | 214 ± 147 | 195 ± 116 | 0.54 | 0.84 | 0.47 | 0.21 | 0.95 | 0.68 |
|  | *Pam3Cys* | 160 ± 120 | 135 ± 97 | 148 ± 110 | 0.56 | 0.89 | 0.18 | 0.36 | 0.55 | 0.35 |
|  | *C. albicans* | 132 ± 93 | 164 ± 125 | 140 ± 111 | 0.42 | 0.79 | 0.28 | 0.34 | 0.88 | 0.70 |
|  | Poly I:C | 35 ± 0 | 35 ± 0 | 35 ± 0 | N/A | N/A | N/A | N/A | N/A | N/A |
| **Adaptive immune system** | | | | | | | | | | |
| IL-17  (pg/ml) | RPMI^c^ | 39 ± 1 | 40 ± 10 | 39 ± 0 | N/A | N/A | N/A | N/A | N/A | N/A |
|  | *C. albicans*^c^ | 191 ± 243 | 319 ± 395 | 221 ± 279 | 0.23 | 0.49 | 0.096 | 0.59 | 0.79 | 0.53 |
|  | E. coli^c^ | 22 (34.9) | 19 (27.5) | 9 (20.0) | 0.36 | 0.25 | 0.36 | 0.71* | 0.095 | 0.71* |
| IL-22  (pg/ml) | RPMI^d^ | 88 ± 27 | 86 ± 30 | 80 ± 10 | N/A | N/A | N/A | N/A | N/A | N/A |
|  | *C. albicans*^d^ | 791 ± 727 | 1265 ± 1466 | 1507 ± 2032 | 0.44 | 0.41 | 0.11 | 0.75 | **0.022** | **0.041** |
|  | E. coli^d^ | 24 (38.7) | 36 (54.5) | 16 (35.6) | 0.051 | **0.028** | 0.074 | 0.090* | 0.74 | 0.85 |
| IFN-γ  (pg/ml) | RPMI^c^ | 24 ± 3 | 25 ± 9 | 24 ± 3 | N/A | N/A | N/A | N/A | N/A | N/A |
|  | *C. albicans*^c^ | 627 ± 1000 | 1101 ± 2097 | 1209 ± 4071 | 0.84 | 0.63 | 0.69 | 0.92 | 0.87 | 0.98 |
|  | E. coli^c^ | 44 (69.8) | 48 (69.6) | 28 (62.2) | 0.42 | 0.096 | 0.97 | 0.44* | 0.41 | 0.54 |

Linear regression was used to analyze cytokine production capacity, while logistic regression was applied for TNF-α and IL-6 (Poly I:C stimulation) and for IL-17, IL-22, and IFN-γ (*E. coli* stimulation). Log10 transformed variables for linear regression are presented in italics. Comparison between AS patients with and without ASCVD and each of the groups to healthy controls was adjusted for age, sex, body mass index (BMI), year and season of blood collection, active smoking status, low-density lipoprotein cholesterol (LDL-C), statin use, glucose, and systolic blood pressure (SBP). Both unadjusted and adjusted p-values are displayed. P-values remaining significant after multiple testing correction are shown in bold. (AS) aortic stenosis, (ASCVD) atherosclerotic cardiovascular disease, (*C. albicans*) *Candida albicans* conidia, (*E. coli*) *Escherichia coli*, (IFN-γ) interferon-γ, (IL) interleukin, (IL-1Ra) interleukin-1 receptor antagonist, (LPS) lipopolysaccharide, (MSU) monosodium urate crystals, (Pam3Cys) tri-palmitoyl-S-glyceryl-cysteine, (PBMC) peripheral blood mononuclear cells, (Poly I:C) polyinosinic:polycytidylic acid, (RPMI) Roswell Park Memorial Institute medium, (TNF-α) tumor necrosis factor-α. ^a^ AS with ASCVD N=70, AS without ASCVD N=44. ^b^ AS with ASCVD N=70, AS without ASCVD N=45. ^c^ AS with ASCVD N=69, AS without ASCVD N=45. ^d^ AS with ASCVD N=66, AS without ASCVD N=45, Healthy controls N=62. * Active smoking status was removed from the adjusted model.

## **Supplemental Table 5. Histological and immunohistochemical analysis of valvular inflammation and remodeling between AS patients with and without ASCVD.**

|  | **Patients with AS (N=46)** | | **Unadjusted  P-value** | **Adjusted  P-value** |
| --- | --- | --- | --- | --- |
|  | **With ASCVD**  **(N=30)** | **Without ASCVD**  **(N=16)** |  |  |
|  | **Mean ± SD** | **Mean ± SD** |  |  |
| Calcification (%) | 29.4 ± 14.6 | 35.8 ± 12.0 | 0.14 | 0.060 |
| Fibrosis (%) | 22.5 ± 7.7 | 21.4 ± 7.8 | 0.64 | 0.37 |
| Elastin (%) | 6.3 ± 4.3 | 6.6 ± 3.3 | 0.80 | 0.94 |
| Proteoglycans (%) | 4.5 ± 5.1 | 4.5 ± 2.9 | 1.00 | 0.64 |
| CD45 no./valve area | 26.78 ± 14.98 | 21.68 ± 11.77 | 0.24 | 0.30 |
| *CD68 no./valve area* | 48.86 ± 37.86 | 29.11 ± 11.73 | 0.036 | 0.052 |
| *CD20 no./valve area* | 1.58 ± 2.29 | 3.54 ± 7.03 | 0.61 | 0.47 |
| CD3 no./valve area | 14.85 ± 7.60 | 18.96 ± 9.39 | 0.12 | 0.12 |
| *MPO no./valve area* | 0.88 ± 1.04 | 1.15 ± 1.99 | 0.90 | 0.92 |

Linear regression was used to compare AS patients with and without ASCVD, adjusting for sex. Log10 transformed variables for linear regression are presented in italics. Both unadjusted and adjusted p-values are displayed. P-values remaining significant after multiple testing correction are shown in bold. (AS) aortic stenosis, (ASCVD) atherosclerotic cardiovascular disease, (CD) cluster of differentiation, (MPO) myeloperoxidase.

# **Supplemental Figures**

**Supplemental Figure 1. Manual gating strategy using flow cytometry.** Doublets and debris were first excluded (A, B), followed by selection of live CD45⁺ leukocytes (C). These leukocytes were then classified into granulocytes or monocytes and lymphocytes (D). From the lymphocyte population, CD19⁺ B cells (E), CD3⁺ T cells (F), and CD56⁺ natural killer (NK) cells (G) were removed. Subsequently, CD16⁺CD56^dim NK cells (H) and CD16⁺CD56^dimHLA-DR⁻ NK cells (I) were excluded from the HLA-DR⁺CD14⁺CD16⁺ monocyte population. Monocytes were then further subdivided into distinct subsets (J–L). The boundary between CD14⁺⁺CD16^dim classical monocytes and CD14⁺⁺CD16⁺ intermediate monocytes was determined based on CD14 and CD16 expression as well as HLA-DR levels (K). The separation between intermediate monocytes and CD14⁺CD16⁺⁺ non-classical monocytes was based on CD14 and CD16 expression in combination with CCR2 levels (L). Finally, expression of the platelet-interaction marker CD41 and monocyte activation markers HLA-DR, CD11c, CD11b, CCR2, and CCR5 was assessed across all monocytes and within individual subsets. Gates for activation markers CD11c, CD11b, CCR2, and CCR5 were defined using the fluorescence-minus-one (FMO) approach (M).^1^

**Circulating inflammatory and functional profiles**

**Circulating inflammatory and functional profiles**

**AS - ASCVD**

**AS + ASCVD**

**Spearman’s rank**

**Valve immunohistology**

**Valve immunohistology**

**Supplemental Figure 2. Correlation patterns of circulating inflammatory and functional profiles and valve phenotypical markers in AS patients with (N=16) and without ASCVD (N=13).** Unsupervised hierarchical clustering heatmaps depict correlations among circulating inflammatory and functional markers and valve phenotypical markers in AS patients with and without ASCVD. Clustering was performed using Spearman’s correlation coefficients, where red indicates positive correlations and blue indicates negative correlations. Correlations with nominal P-values < 0.05 are marked with an asterisk (*). (AS) aortic stenosis, (ASCVD) atherosclerotic cardiovascular disease.

# **References**

1.	Feher K, Kirsch J, Radbruch A, Chang HD, Kaiser T. Cell population identification using fluorescence-minus-one controls with a one-class classifying algorithm. *Bioinformatics*. 2014;30(23):3372-8.10.1093/bioinformatics/btu575.
